# Support Structures Optimisation for High-Quality Metal Additive Manufacturing with Laser Powder Bed Fusion: A Numerical Simulation Study

**DOI:** 10.3390/ma16227164

**Published:** 2023-11-14

**Authors:** Antonios Dimopoulos, Mohamad Salimi, Tat-Hean Gan, Panagiotis Chatzakos

**Affiliations:** 1 Department of Mechanical and Aerospace Engineering, Brunel University London, Uxbridge UB8 3PH, UK; tat-hean.gan@brunel.ac.uk; 2 Brunel Innovation Centre, Brunel University London, Uxbridge UB8 3PH, UK; mohamad.salimi@brunel.ac.uk; 3 TWI Ltd., Granta Park, Great Abington, Cambridge CB21 6AL, UK; 4 TWI Hellas, Leof. Kifisias 280, 152 32 Chalandri, Greece; panagiotis.chatzakos@twi.gr

**Keywords:** additive manufacturing, numerical modelling, thermo-mechanical analysis, metal support structures, multi-objective optimisation, laser powder bed fusion, titanium alloy

## Abstract

This study focuses on Metal Additive Manufacturing (AM), an emerging method known for its ability to create lightweight components and intricate designs. However, Laser Powder Bed Fusion (LPBF), a prominent AM technique, faces a major challenge due to the development of high residual stress, resulting in flawed parts and printing failures. The study’s goal was to assess the thermal behaviour of different support structures and optimised designs to reduce the support volume and residual stress while ensuring high-quality prints. To explore this, L-shaped specimens were printed using block-type support structures through an LPBF machine. This process was subsequently validated through numerical simulations, which were in alignment with experimental observations. In addition to block-type support structures, line, contour, and cone supports were examined numerically to identify the optimal solutions that minimise the support volume and residual stress while maintaining high-quality prints. The optimisation approach was based on the Design of Experiments (DOE) methodology and multi-objective optimisation. The findings revealed that block supports exhibited excellent thermal behaviour. High-density supports outperformed low-density alternatives in temperature distribution, while cone-type supports were more susceptible to warping. These insights provide valuable guidance for improving the metal AM and LPBF processes, enabling their broader use in industries like aerospace, medical, defence, and automotive.

## 1. Introduction

Over the past years, Additive Manufacturing (AM), also known as 3D printing, has revolutionised the manufacturing industry, from developing concepts to producing complex, thin, lightweight, and fully functional parts [1]. The joint ISO/ASTM terminology standard defines AM as the “process of joining materials to make parts from 3D model data, usually layer upon layer, as opposed to subtractive manufacturing and formative manufacturing methodologies” [2]. As the use of AM technologies reduced the time necessary to make prototypes, these technologies were often referred to as “rapid prototyping” and the possibility of using such techniques as a manufacturing process for metal parts became highly appealing [3].

Laser Powder Bed Fusion (LPBF) is one of the most promising AM technologies and can be directly used to fabricate metal components with high precision and performance for various industries such as aerospace, biomedical, defence, and automotive [1,4]. In LPBF, a laser beam selectively melts the powder in a powder bed, several melting tracks are strung together in a micro-welding process, and a 3D component is created inside the powder envelope where several of these layers are fused together [5]. Various materials can be used in LPBF, such as aluminium, copper, nickel, and titanium, while the mechanical properties of the produced parts can be similar to or even better than those of parts manufactured via traditional methods, such as machining and moulding [1,5].

However, the serious weaknesses of this technology include the construction of overhang structures and the residual stress inherent in the melting and solidification process [6]. In LPBF, the material in the build is melted and cooled several times, and stress is accumulated due to inconsistent levels of heating [7]. This residual stress leads to severe problems because it can cause warpage, cracks, and delamination during processing, which may block the recoater blade and result in a build failure [8]. It could be assumed that the unmelted powder around the part is able to support the overhang surfaces and reduce the thermal stresses; however, Poyraz et al. [9] and Bo and Chou [10] found that this was not feasible since the unmelted powder is not thermally conductive. Thus, support structures are always required, since they anchor the part to the build plate, offer a suitable platform for the next layer to be built upon, and act as a heat sink that allows the part to cool at a more controlled rate. Therefore, producing an object without or insufficient support structures results in distorted and collapsed parts, while the addition of unnecessary supports increases the post-processing, the time and effort needed to remove them, the risk of damaging the part, and the amount of material required [11].

In the realm of mechanics and thermodynamics, thermal stress pertains to the mechanical stress generated by alterations in a material’s temperature. To delve into the behaviour of materials during LPBF, a potent tool is Thermal Mechanical Analysis (TMA). TMA is a technique designed to gauge a material’s dimensional changes under carefully controlled heating and cooling conditions, thereby offering valuable insights into both the thermal and mechanical properties of the material [12]. When the cumulative thermal stress exceeds the material’s yield stress, it can induce fracturing or plastic deformation. In the context of LPBF, when a hot layer is deposited, the lower portion typically makes contact with a metal surface, while the upper layer is surrounded by air. The faster cooling of the top layer compared to the bottom layer can result in shrinkage at the top layer, primarily due to differing thermal diffusivities between the metal and air [13,14].

Several recent studies have used TMA to investigate the thermal expansion and deformation behaviour of materials during LPBF. Chen et al. [4] developed a layer-by-layer model to examine the residual stress of the typical sections during the LPBF processes. Their investigation identified lower residual stress for hollow and semi-hollow parts compared with solid parts. Further suggestions, such as using a rounded corner instead of a sharp one or an arc structure instead of a straight one, were proposed to reduce stress in LPBF. This is due to the fact that sharp edges normally cool quicker than the centre. Dai et al. [15] developed an Ansys model to predict the thermal history and warpage of a layer-by-layer build part in LPBF processes. It was found that changing from the initial solid elements to powder elements results in higher temperature gradients, larger transient and residual stresses, and increased warpage. Javid and Ghoreishi [16] analysed the thermal deformation behaviour of Inconel 718 alloy during LPFB using TMA. It was found that the deformation behaviour was related to the micro-structure of the material and was influenced by the heating rate. Yang et al. [17] investigated the thermal deformation behaviour of 17-4PH stainless steel during LPFB using TMA. The study found that the material experienced significant deformation during the process, and the deformation behaviour was influenced by the scanning speed and laser power. Mugwagwa et al. [18] presented a thorough analysis of stress relief heat treatment techniques. The effectiveness of stress relief using an in situ annealing method was reported by Edin et al. [19] and a technique based on Barkhausen noise analysis was proposed by Staub et al. [20] to measure residual stress in LPBF. TMA can also provide insights into the mechanical properties of materials during LPFB. Knezevic et al. [21] investigated the effects of porosity on the thermo-mechanical behaviour of Ti-6Al-4V alloy during LPFB using TMA. It was found that the mechanical properties of the material were improved with increasing porosity, and the thermal expansion behaviour was affected by the porosity level. Cheng et al. [8] investigated the feasibility of using topology optimisation to design support structures to mitigate residual stress and build failures. They exploited the inherent strain method to perform fast prediction of residual stress in an additive manufacturing build. The design of the support structure utilises graded lattice structures, taking advantage of their open-celled and self-supporting characteristics. The optimisation objective was to minimise the mass of the sacrificial support structure while adhering to stress constraints. By limiting the maximum stress to below the yield strength, the occurrence of cracking resulting from residual stress can be avoided. To demonstrate the feasibility of the proposed approach, support structures for a double-cantilever beam and a hip implant were designed. After optimisation, the weight of the support structure was reduced by approximately 60%. The optimised support structures exhibited no stress-induced cracking when the designs were implemented through AM, thus confirming the effectiveness of the proposed method. Adams and Peppiatt [22] showed that, at low temperatures, using a combination of internal tapering and fillet can mitigate the role of thermal stresses in joint failure. A thermo-mechanical phase-field fracture model was developed by Raun et al. [23] to simulate hot cracking in additive manufacturing. It was observed that the circumferential crack formation is mainly due to the solidification shrinkage strain, while the central crack is related to the temperature gradient. To remove hot cracking from aluminium alloy 6061, Opprecht et al. [24] suggested adding various quantities of Yttrium Stabilised Zirconia (YSZ) to the aluminium alloy powder using a dry mixing (Turbula) procedure. Burkhardt et al. [25] analysed the effectiveness of various thermo-mechanical simulations for laser powder bed fusion. Such an analysis is required to predict the residual stress and deformation associated with the temperature associated with the welding concepts [26,27]. In welding, however, the material undergoes fewer cycles of heating and cooling compared with the LPBF procedure. In a fully coupled thermo-mechanical mesoscale model for LPBF, the laser beam is considered as a moving body heat source [28,29]. To reduce the computational time, the simulation is carried out on spots, tracks, or layers followed by applying some simplifications [30,31,32,33]. By neglecting the heat loss effect and assuming homogeneous and isotropic materials, Ma et al. [31] developed an FE model to predict surface melting and solidification due to a single laser pulse. Nickel et al. [34] used a three-dimensional FE model to predict thermal stress and deformation due to deposition patterns. Excluding the surrounding powder or the use of geometrically linearised material models in the numerical modelling causes inaccuracies in the prediction [35,36]. Lastly, Zaeh F. and Branner G. [37] developed a numerical simulation based on finite element analysis (FEA) to evaluate and quantify the resulting residual stresses and deformations due to the temperature gradient mechanism (TGM). The investigations focused on coupled thermo-mechanical models incorporating specific boundary conditions and temperature-dependent material properties to identify the heat impact on residual stresses and deformations for LPBF systems.

Based on the literature referenced above, much work has been conducted to predict and evaluate the thermo-mechanical behaviour of metal parts using FEA or similar advanced models and techniques. Either way, it is clear that, to establish LPBF in production technology, extensive knowledge about the transient physical effects during the manufacturing process is mandatory [37]. However, the investigation of support structures’ performance, considering supports and the overhang part as a single assembly, is very limited.

In this research, for the first time, four support types were investigated, conducting 3D printing and computer-aided thermo-mechanical simulations, for the proposal of optimised support geometries that better transmit the heat while maintaining a high quality of the printed part. The optimisation approach focused on the evaluation of the various geometric support parameters for block, line, contour, and cone support structures using the design of experiment methodologies and relevant optimisation algorithms. Based on the findings, along with previously published research conducted by the author [11], optimised parameters that generate low-volume and easily removed supports, without significantly affecting the quality of the part, are proposed.

## 2. Experimental Study for Block Support Structures

In previously published work [11], the author evaluated the various support and process parameters for metal LPBF by 3D printing and testing small specimens to propose optimised support structures that minimise the support volume, support removal effort, and warping deformation. A similar approach is proposed in this research via 3D printing small L-shaped specimens supported by block-type support structures. Along with the 3D printings, a computer-aided thermo-mechanical analysis on existing support structures was conducted, not only to evaluate the performance of the various support geometries in terms of the heat transmission and distortion caused by thermal stresses, but also to investigate the behaviour of the part and the supports while 3D printing and when exposed to high laser temperatures.

Thus, to investigate the behaviour of supported overhang surfaces, small ledge specimens were 3D printed using Selective Laser Melting (SLM) technology, and block-type support structures with configurable geometric parameters were evaluated. For the needs of the experiments, an EOS M290 machine with a Yb-fibre laser of 400 W and a focus diameter of 100 μm was used for fabricating the specimens. The material used was the EOS Titanium Ti64 Grade 5 in powder form: a strong and lightweight Ti6Al4V alloy with a generic particle size distribution of 20–80 μm, and a powder chemical composition of Ti (balance), AI (5.50–6.75 wt%), V (3.50–4.50 wt%), and 1.05 wt% of other elements.

**Figure 1:**
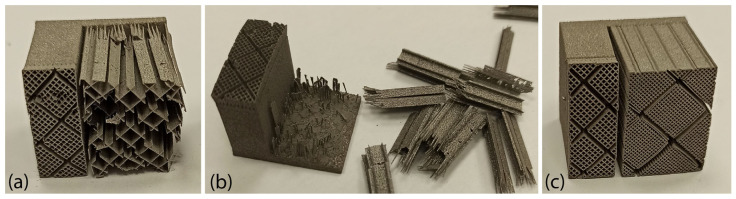
Screening experiments to evaluate the various support parameters: (**a**) distorted low-density supports; (**b**) easy to remove low-density supports; (**c**) warped high-density supports.

The aim of these experiments was to define the minimum and the maximum support parameters that best produce the specimens without significant printing defects and build failures. It was found that low dense support structures were more prone to distort and crack due to high thermal stresses applied in the area, as shown in Figure 1a. In addition, the majority of low-density supports resulted in collapsed parts and build failures due to insufficient material being used to support the overhang surface; however, they were very easy to remove as illustrated in Figure 1b. On the other hand, high-density support structures, illustrated in Figure 1c, were less prone to warp and distort, while most of the specimens were printed successfully. However, their removability was much more difficult compared to low-density supports.

**Figure 2:**
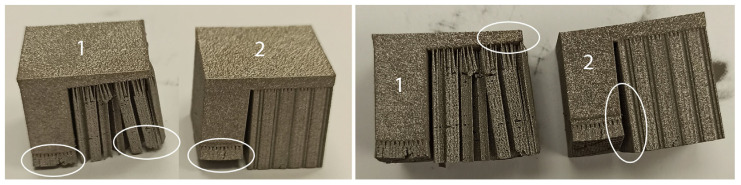
Warped low-density “1” and high-density “2” ledge specimens fabricated using SLM.

Moreover, due to the high temperature and thermal stresses applied while printing, it was observed that both high- and low-density support structures had significant effects on the quality of the printed specimen. Specifically, as highlighted in Figure 2, the region surrounding the build plate emerged as the most critical area, experiencing the most pronounced warping deformations. Additionally, geometric assessments revealed substantial distortions in the overhang surfaces of the majority of printed specimens, particularly when low-density support structures were used.

Given the findings mentioned earlier, it became crucial to undertake numerical investigations into the thermo-mechanical responses of the typical support structures utilised in metal AM and LPBF. The objective was to suggest improved support configurations that enhance print quality, even in the face of the extreme thermal conditions during printing. To achieve this, a set of numerical simulations was carried out to cross-reference the results with the experimental data. Initially, the study focused on analysing block support structures, and subsequently, line, contour, and cone structures were investigated in the later stages of the research.

## 3. Numerical Simulations

### 3.1. Specimen and Areas of Investigation

**Figure 3:**
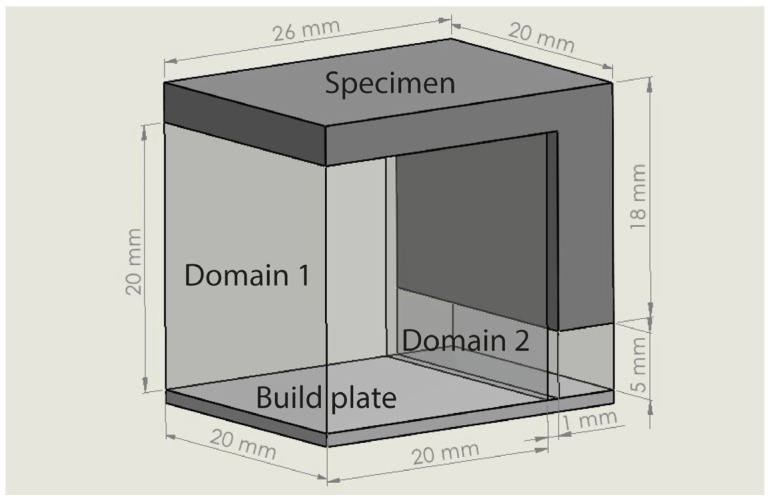
L-shaped specimen and support structure design domains.

To validate the experimental results, a thermo-mechanical simulation was created using COMSOL Multiphysics and a small L-shaped specimen was designed. As observed in the literature, such small ledge overhang geometries are most commonly used in LPBF and metal AM to test and evaluate the performance of the support structures. The specimen’s geometry along with its dimensions and the supports’ design domains are illustrated in Figure 3. The two domains where support structures were generated are highlighted in light grey (transparent mode), while the L-shaped specimen is in dark grey. Domain 1 (20 × 20 × 20 mm) supports the bigger ledge overhang, while domain 2 (20 × 5 × 5 mm) supports the smaller overhang, which first anchors the specimen to the built plate. Underneath the two domains, a part of the build plate is also illustrated. In this study, only the performance of the supports in domain 1 was investigated. Domain 2 remained in a fully dense support volume for every support alternative.

### 3.2. Numerical Modelling and Scene Setup

Before initiating the thermo-mechanical simulations on the component with the block-type support, preliminary experiments were carried out to establish the primary framework and identify the specific areas for investigation in terms of the thermal and mechanical analysis. The goal was to simulate the LPBF process as much as feasible and record the thermo-mechanical behaviour of the imported assembly. Titanium alloy was used as a material and a heat source of 1550 °C, which is approximately the melting point of titanium, was set between the supports and the part’s overhang surface.

All the simulations were performed in COMSOL Multiphysics v6.1, while the 3D models were designed in SolidWorks, San Diego, CA, USA. The integration of specimens and supports from SolidWorks to COMSOL is a straightforward process. This seamless transfer allows for a smooth transition between the two software platforms, enabling users to effectively utilise the features and capabilities of both programs. By importing parts and support from SolidWorks to COMSOL, users can leverage the advanced modelling and simulation capabilities of COMSOL while benefiting from the design and engineering functionalities of SolidWorks. This integration enhances the overall efficiency and effectiveness of the design and analysis process, enabling users to seamlessly work with complex models and simulations.

Various materials can be used in metal AM and LPBF systems such as steels (Fe + C +…), aluminium-based alloys (Al + Si + Mg), titanium-based alloys (Ti + Al +…), and nickel-based alloys (Ni + Cr +…). The choice depends on the application, the material properties, and the compatible 3D printing machine. In this study, a grade 4 titanium was selected since its properties are very close to the majority of the titanium alloys used in LBPF and metal AM in general. It is a strong and lightweight Ti alloy with excellent corrosion resistance, a max melting temperature of 1660 °C, and a specific heat capacity of 0.53 (J/g) °C. In the modelling, the modulus of elasticity was assumed to be 105 GPa with a Poisson’s ratio of 0.37 and the thermal conductivity was assumed to be 17.2 W/mK.

Regarding the heat source, a value of 1550 °C was set, which represents the melting point of titanium while printing in SLM (usually between 1500 °Cand 1660 °C [5,38]). The amount of energy transferred from the heating source to the powder is directly linked to the laser power being used. When the laser power is set at a specific value, such as 400 W for a volume of 1 mm³, the total power applied to the powder will correspond to this value. With this laser power, the powder undergoes melting, causing temperatures to exceed 1550 °C. Nevertheless, it is important to note that the primary focus of this research was to investigate the heat transfer process from the component to the support structure after the phase transition of the powder from a molten liquid to a solid state. This correlation is crucial as it guarantees that the heating source effectively delivers the intended energy to the powder, enabling precise management and manipulation of the thermal effects throughout the process. By ensuring that the heating source energy aligns with the actual laser power, the system can attain dependable and precise outcomes in terms of temperature control and material transformation. This synchronisation between the heating source and laser power is essential for achieving consistent and accurate results in the overall process.

To demonstrate the thermal stress, heat distribution, temperature spectrum, and heat transmission from one domain to another, various branches of physics, such as heat transfer in solids and solid mechanics, were employed. These disciplines were utilised to analyse thermal behaviour and illustrate how heat was transferred within a system, including support structures. The analysis revealed both the thermal stress and heat distribution on the part under consideration. To maintain simplicity and focus on the main assembly, the metal powder surrounding the part was excluded from the simulations.

In thermo-mechanical modelling, a significant aspect involves examining thermal expansion, which refers to the material’s tendency to alter its volume in response to temperature variations. Within this modelling framework, heat transfer is calculated alongside structural mechanics, which is treated as a combined problem. Throughout the modelling process, it was assumed that the surface of the component in contact with the support maintained a temperature of 1550 °C. Consequently, it became feasible to compute the thermal expansion (ε_inel_) linked to that specific layer using the formula: $\varepsilon_{inel}=\alpha (T-T_{ref})$. Here, “α” represents the coefficient of thermal expansion, “T” denotes the known applied temperature, and “T_ref_” corresponds to the temperature at which zero thermal stress occurs. The coefficient of thermal expansion was considered to be 8.6 × 10^−6^ (°C)^−1^ and the temperature applied “T” was established as 1550 °C [39].

Heat dissipation was also considered, and it was presumed that the entire model was surrounded by a temperature of 21 °C (T_ref_). Consequently, the heat loss at this location arose from both heat transmission from the support and radiation to the surrounding environment. The modelling process encompassed two boundary conditions: one pertained to the build plate, while the other related to the point where the component connected with the smaller part in contact with the build plate. The supports and the part were made to be uniform to emphasise the contrast more effectively in their heat transfer capabilities.

### 3.3. Overview of the Thermo-mechanical Simulation

**Figure 4:**
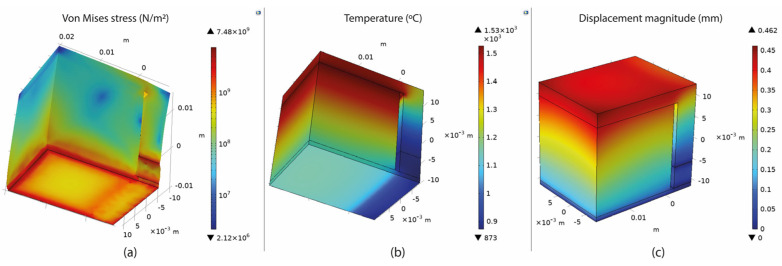
Fully dense supports: (**a**) thermal stress; (**b**) temperature distribution; (**c**) displacement.

Based on the setup mentioned in Section 3, numerical simulations of fully dense supports (see Figure 3) were conducted to provide a better understanding of the heat transmission from the part to the supports. As shown in Figure 4a, high thermal stresses occurred in the build plate, especially on the edges around the supports. This can cause warping, distortion, or, even worse, it can detach the part from the build plate while printing. As observed in Section 2, due to high thermal stresses, the short supports that anchored the part to the build plate could become significantly warped, while the supports underneath the overhang surface could become cracked. Concerning the distribution of temperatures (refer to Figure 4b), in this specific simulation configuration, it was observed that the peak temperatures were concentrated near the heat source, situated between the supporting structures and the overhang surface of the component. It can also be observed that high temperatures were developed along the whole support body up to the build plate; however, this temperature distribution depends on the morphology of the existing support structures in each case. Regarding the plot showing the magnitude of displacement (see Figure 4c), it was observed that the greatest deformation took place on the overhang surface of the component, particularly at the front edge. Similar results were also noticed during the screening experiments of Figure 2, where the geometric verification showed that the front edge of the overhang surface was significantly distorted from its actual position. The stress distribution arises from thermal expansion, and because we set a boundary condition at the base plate to restrict displacement, this resulted in the observation of higher stress values.

### 3.4. Methodology and Numerical Simulations for Block Supports

In LPBF, the scanning speed and laser power are two of the most important parameters, which significantly affect not just the ease of removing supports in post-processing, but also the deformation behaviour of the printed part [17,40]. In this study, as mentioned in Section 3.2, to perform the thermo-mechanical simulations, laser power was considered as the main heat source while various support types along with their parameters were investigated. There is a wide range of support types available in LPBF and their choice depends on the geometry and the features of the part, the selected material, and the compatible slicing software [9,41,42]. According to the literature, block, line, contour, and cone supports are the most commonly used support structures in LPBF; consequently, our study focused on investigating these specific types of support structures.

**Figure 5:**
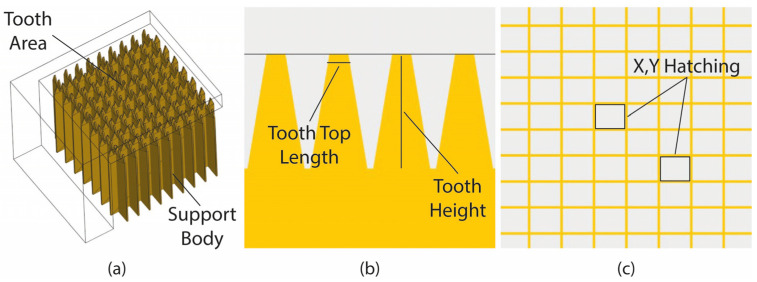
(**a**) Morphology of block supports; (**b**) tooth area; (**c**) grid density.

In this section, block-type supports are investigated with the aim of showing the phenomena observed in the experimental study. Their morphology associated with the supports is shown in Figure 5. It was observed that they can be divided into two areas: (i) the support body, which defines the geometry and the density of the supports; and (ii) the tooth area which is based on the contact points between the supports and the part’s overhang surface (Figure 5a).

The next step was to define the input parameters (free variables that can be changed and controlled) to be investigated along with their respective levels. Based on the literature and the author’s prior publication [11], three main geometric parameters were selected for investigation: tooth height, tooth top length, and X, Y hatching (Figure 5b,c). Table 1 illustrates the selected parameters along with their respective levels. Each parameter contains three levels: the minimum, the average, and the maximum value.

During the Design of Experiments (DOE), the Response Surface Methodology (RSM) based on Central Composite Design (CCD) was used to perform the experiments and define the different configurations. This method was selected since it is especially useful in the analysis, visualisation, and optimisation of responses. Regarding the CCD setup, a face-centred approach was followed with an alpha value equal to 1, while no replicates of factorial, axial, and centre points were used. As a result, 15 unique alternatives (8 factorial, 6 axial, and 1 centre) for this investigation were selected (see the Supplementary Material files). All the DOEs, the data analysis, the visualisation, and the optimisation of the responses were performed in the Design-Exert v13 software.

**Figure 6:**
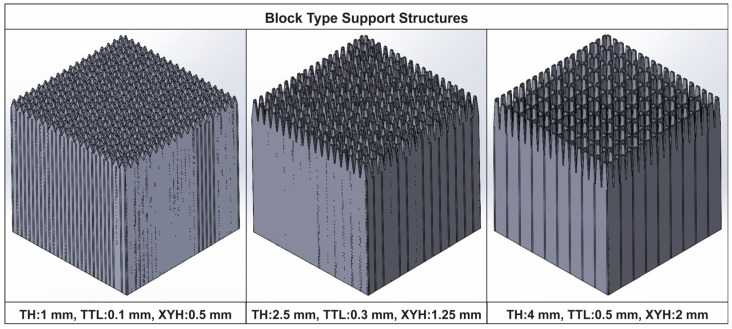
Sample of block support alternatives.

Thus, for the DOE for block-type supports, the CAD assembly used to set up and run the simulations is shown in Figure 3. The L-shaped specimen, support domain 2, and the build plate area were kept intact; while in domain 1, the various support alternatives that arose during the DOE were thermo-mechanically analysed one by one. A sample of the designed block support structures along with some of their input parameters are illustrated in Figure 6. As a result, a total of 15 simulations were performed.

### 3.5. Performance Measures

After the completion of the thermo-mechanical simulations, in order to evaluate the performance of the support structures, four responses were investigated: support volume, thermal stress, plate temperature, and overhang displacement. The volume of the support structures was measured in SolidWorks. Then, they were exported in STEP format and imported one by one in COMSOL where the simulations were conducted.

**Figure 7:**
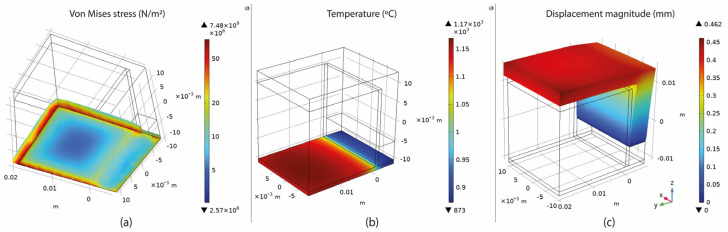
(**a**) Stresses on the build plate; (**b**) temperature on the build plate; (**c**) part deformation.

The thermal stress was measured by recording the maximum value (calculated in COMSOL) of the overall stresses applied on the build plate since it was found that the highest values of thermal stress occurred around the build plate where the supports were anchored (Figure 4a). Thus, this area was further investigated since it was considered the most critical point for developing warping and distortion defects. For better visualising the results in COMSOL software, an algorithmic scale was preferred (Figure 7a).

The temperature distribution was measured by recording the maximum value of the build plate temperature calculated in COMSOL since the overall approach was to investigate the heat transmission and the heat capacity of the various support structures. As shown in Figure 7b, higher temperatures were observed on top of the build plate where the support structures were anchored. Here, it should be noticed that thermal stress and temperature distribution were strongly connected to each other and both significantly affected not only the printed part but also the supports. Based on the results, their correlation is further explained in the discussion section.

The displacement magnitude was measured in terms of the maximum deformation value of the assembly based again on the COMSOL simulations. It was found that the maximum deformation occurred on the specimen’s overhang surface with a positive direction on the z-axis, especially on the edge in front, as illustrated in Figure 7c. After recording all the measurements, the values were imported into Design-Expert 13, where Analysis of Variance (ANOVA) was performed for the validation of the selected models, the analysis of the data, the visualisation, and the optimisation of the results.

### 3.6. Results for Block Supports

For the in-depth investigation of block-type supports and their parameters, quadratic models were used to analyse the experiments. According to the ANOVA results, all the factors satisfied the criteria for a well-designed model. Specifically, “F” and “P” values imply that a model and the model terms, respectively, are significant, predicted R² and adjusted R² (both almost equal to 1) indicate a reasonable model agreement, while an Adeq precision (which measures the signal-to-noise ratio) greater than 4 is desirable. Therefore, all the quadratic models were approved for further analysis (see also the Supplementary Material files where the ANOVA results are presented).

**Figure 8:**
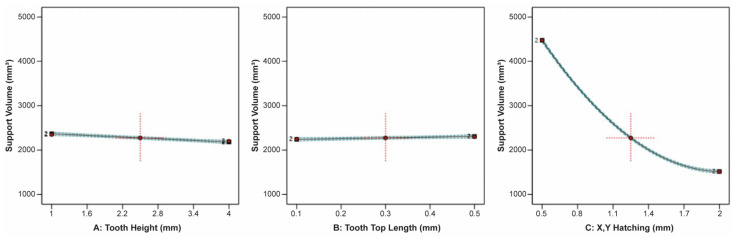
Main plots of support volume in Design-Expert 13 for block-type supports.

The ANOVA showed that tooth height (A), tooth top length (B), and X, Y hatching (C), along with their interactions, had a significant effect on the support volume, thermal stress, plate temperature, and overhang displacement. The correlation between tooth height, tooth top length, and X, Y hatching on the support volume is illustrated clearly in Figure 8. It was found that tooth height and tooth top length barely affected the support volume, while X, Y hatching had the most significant effect by far, since, as the hatching distance increased, the support volume decreased significantly. This is because a significant amount of material is removed from the support structure’s main body as the total number of inner grid walls decreases.

**Figure 9:**
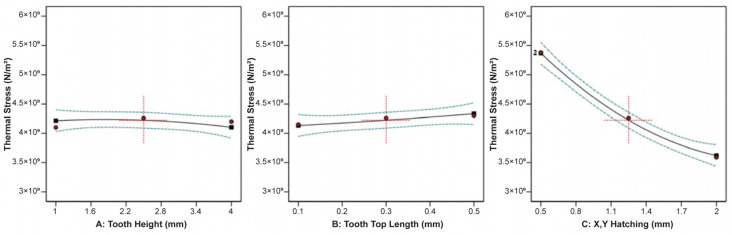
Main plots of thermal stress in Design-Expert 13 for block-type supports.

**Figure 10:**
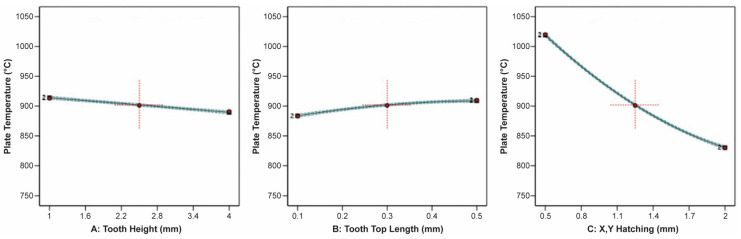
Main plots of plate temperature in Design-Expert 13 for block-type supports.

The effect of tooth height, tooth top length, and X, Y hatching on the thermal stress applied on the build plate is shown in Figure 9. It can be observed that tooth height barely affected the thermal stress, while as the tooth top length increased, the thermal stress slightly increased. On the other hand, as X, Y hatching increased, the thermal stress greatly decreased. Similarities were found in the correlation between the impact of tooth height, tooth top length, and X, Y hatching on the plate temperature since these two responses were strongly connected to each other. As illustrated in Figure 10, it was found that as the tooth height increased, the plate temperature slightly decreased, while as the tooth top length increased, the plate temperature slightly increased. In addition, the X, Y hatching had the most significant effect since as the hatching distance increased, the plate temperature decreased significantly.

**Figure 11:**
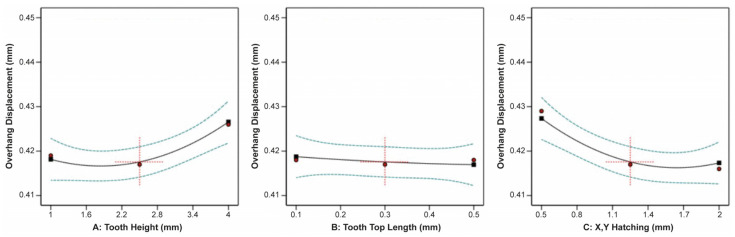
Main plots of overhang displacement in Design-Expert 13 for block-type supports.

The effect of tooth height, tooth top length, and X, Y hatching on the specimen’s overhang displacement is illustrated in Figure 11. It was observed that tooth height and X, Y hatching had the most significant effect on the overhang displacement, while the tooth top length barely affected the displacement. Thus, as the tooth height increased, the overhang displacement increased as well, while as the X, Y hatching increased, the overhang displacement greatly decreased.

### 3.7. Comparison with the Experimental Work

Comparing the numerical simulation findings with the experimental work discussed at the beginning of this article, significant similarities were observed. The numerical simulations showed that the highest thermal stress was applied on the build plate as illustrated in Figure 4a, where the part and the supports were more prone to warp according to the experiments shown in Figure 2. In addition, the highest displacement was observed between the supports and the part’s overhang surface where the highest temperatures were applied (see Figure 4c) resulting in defective and warped overhangs (see Figure 2). On the other hand, based on the criteria that better satisfy the performance measures, the plots and the numerical optimisation results showed that low values of tooth height, average values of tooth top length, and average to high values of X, Y hatching resulted in optimised block support structures, which were able to minimise the risk of defective parts and build failures. Further analyses and comparisons regarding the optimal solutions are recorded in Section 5.4, where a numerical optimisation for block, line, contour, and cone support structures is presented.

## 4. Numerical Simulations for Various Forms of Support Structures

### 4.1. Support Types and Parameters

**Figure 12:**
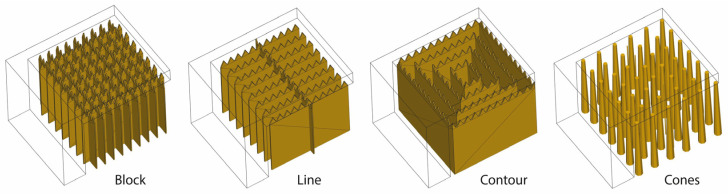
Morphology of block, line, contour, and cone support structures.

As the numerical results for the block-type support were validated against the experimental data, additional numerical simulations were conducted using the same methodology for line, contour, and cone supports, as they are commonly employed in laser powder bed fusion. Line and contour supports share a resemblance with block supports as they both comprise (i) the supporting body, which determines the support’s shape and density, and (ii) the tooth area, which relies on the points of contact between the supports and the overhanging surface of the part. In contrast, cone-type supports consist of individual pillars with adjustable lower and upper diameters. The morphology of block, line, contour, and cone supports is shown in Figure 12.

### 4.2. Methodology and Numerical Simulations for Line, Contour, and Cone Supports

**Figure 13:**
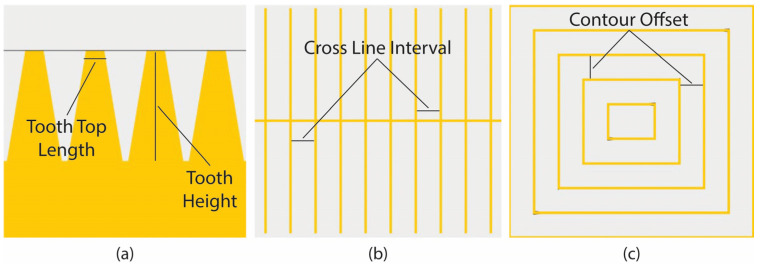
(**a**) Tooth area, (**b**) Cross line interval for line type, (**c**) Contour offset for contour type.

**Figure 14:**
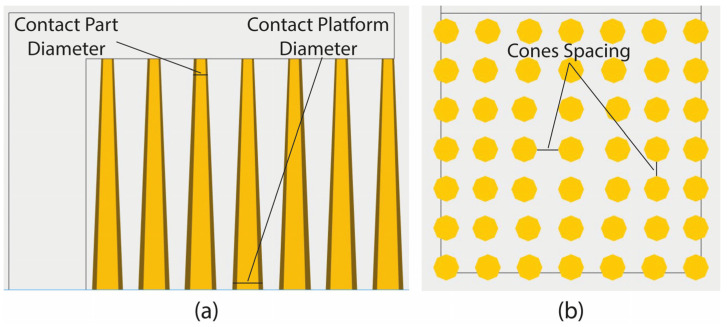
(**a**) Cone-type side view; (**b**) cone spacing for cone-type support.

To perform the DOE for line, contour, and cone supports, similar to block-type supports, three main geometric parameters were selected for investigation: line-type—tooth height, tooth top length, and cross line interval (Figure 13a,b); contour-type—tooth height, tooth top length, and contour offset (Figure 13a,c); cone-type—contact platform diameter, contact part diameter, and cone spacing (Figure 14). Across various types, the areas under examination share similarities as they are all defined by the overall density of the supports and the points of contact between the part and the supports. For block, line, and contour supports, identical input parameters were applied, while a proportionate approach was adopted for cone supports in determining the spacing between the cones and the contact points. Table 2 illustrates the parameters mentioned above for each of the three support types along with their respective levels. Similar to block supports, each parameter contains three levels: the minimum, the average, and the maximum value.

It is important to note that all chosen support types can be created using commonly available slicer software, such as Materialise Magics. However, for the purposes of this study, the supports were designed from the ground up to ensure a parametric design approach, thus minimising the risk of structural problems during simulations. Additionally, beyond the specific parameters investigated in this study, each support type possesses a multitude of other parameters that significantly influence their geometry and ease of removal. These parameters were kept consistent, with their values drawn from the existing literature and relevant experimental research [11,40]. For instance, line and contour supports (as with block supports, which were investigated earlier in this research) were constructed with a 0.2 mm wall thickness, 0.1 mm tooth base interval, and 1 mm tooth base length, while no perforations, fragmentation, or separation width were applied.

**Figure 15:**
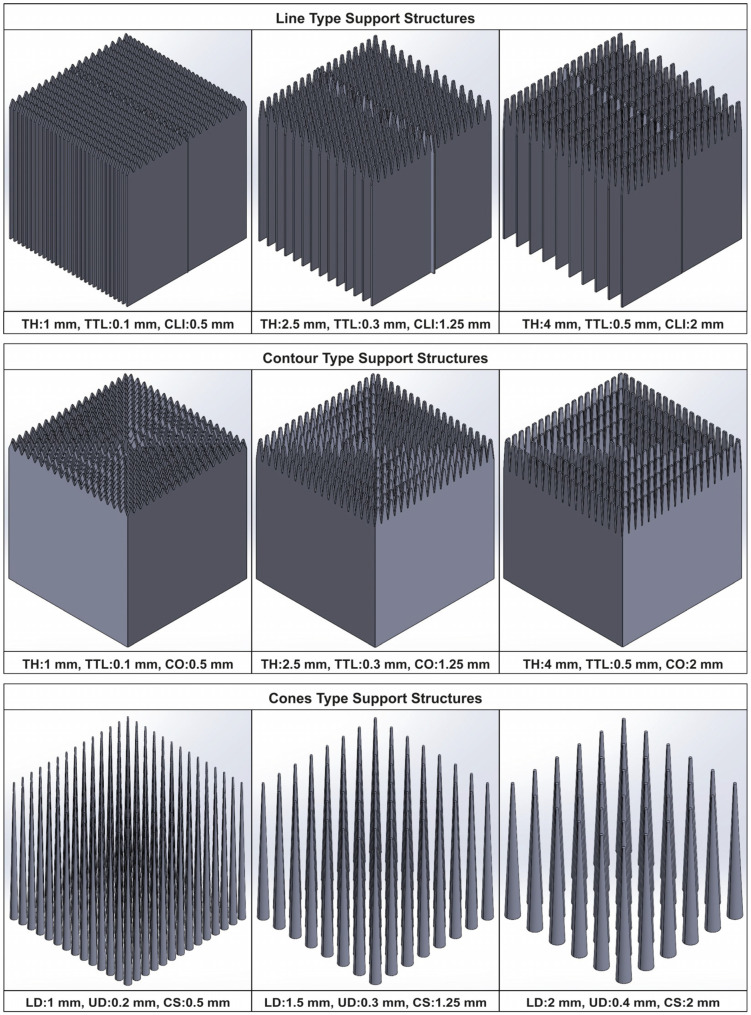
Sample of line, contour, and cone support alternatives.

Similar to the block-type supports, the identical simulation configuration was applied to conduct the thermo-mechanical analysis for line, contour, and cone support structures. The L-shaped specimen, support domain 2, and the build plate area remained unchanged. However, in domain 1, the different support options used during the Design of Experiments (DOE) were individually subjected to thermo-mechanical analysis. An example of the designed line, contour, and cone support structures along with some of their input parameters are illustrated in Figure 15. As a result, a total of 45 simulations were performed. Moreover, the same responses were measured and evaluated as described in Section 3.5. These are the support volume, thermal stress, plate temperature, and overhang displacement.

## 5. Results for Line, Contour, and Cone Supports

### 5.1. Line Type

**Figure 16:**
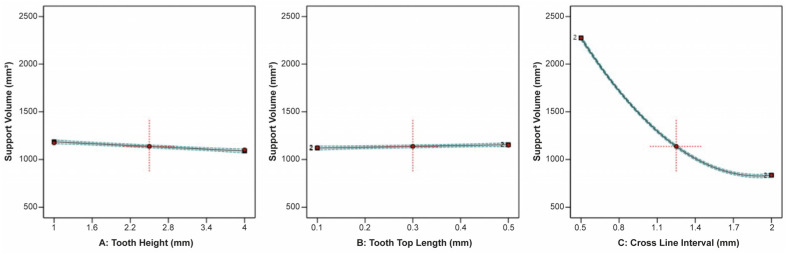
Main plots of support volume in Design-Expert 13 for line-type supports.

For the line-type support structures, the effect of tooth height, tooth top length, and cross line interval on the support volume is illustrated in Figure 16. Similar to the block-type supports, it was observed that the tooth height and tooth top length had a minimal impact on the support volume, whereas the cross line interval had a significant effect since, as the cross line interval increased, there was a substantial decrease in the support volume.

**Figure 17:**
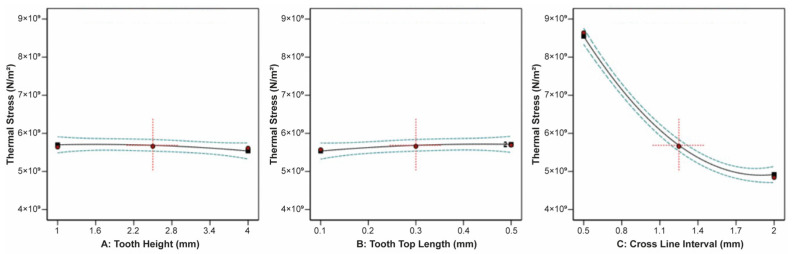
Main plots of thermal stress in Design-Expert 13 for line-type supports.

**Figure 18:**
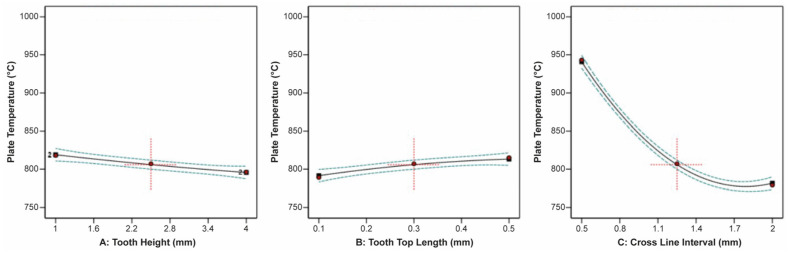
Main plots of plate temperature in Design-Expert 13 for line-type supports.

The correlation between the tooth height, tooth top length, and cross line interval on the thermal stress and temperature applied on the build plate are shown in Figure 17 and Figure 18, respectively. It can be observed that the tooth height and tooth top length had a minimal impact on the thermal stress, whereas increasing the cross line interval led to a significant reduction in the thermal stress. On the other hand, tooth height and tooth top length only slightly influenced the plate temperature. Specifically, as the tooth height increased, the plate temperature marginally decreased, and as the tooth top length increased, the plate temperature slightly increased. Cross line interval exerted a pronounced influence on the plate temperature, with an extreme decrease as the cross line interval increased.

**Figure 19:**
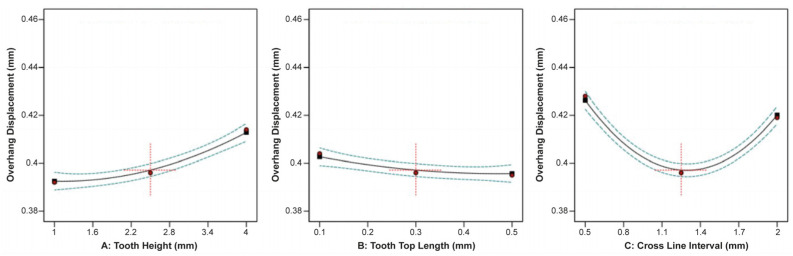
Main plots of overhang displacement in Design-Expert 13 for line-type supports.

The ANOVA results regarding the overhand displacement and its correlation with the tooth height, tooth top length, and cross line interval are shown in Figure 19. It was found that an increase in the tooth height led to a corresponding increase in the overhang displacement. Conversely, an increase in the tooth top length resulted in a slight decrease in the overhang displacement. However, it is important to note that this support type exhibited a unique behaviour. In the cross line interval plot, an increase in the cross line interval led to a decrease in the overhang displacement within a range from 0.5 mm to 1.1 mm, while conversely, it increased within a range from 1.4 mm to 2 mm. This phenomenon may be attributed to the distinctive geometry of the line-type support structures.

### 5.2. Contour Type

**Figure 20:**
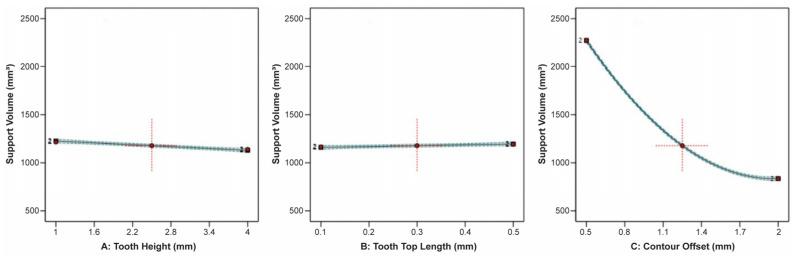
Main plots of support volume in Design-Expert 13 for contour-type supports.

Regarding the contour-type support structures, the findings were also very close to those of the block-type and line-type supports. It was observed that tooth height and tooth top length did not significantly affect the support volume, as illustrated in Figure 20. On the contrary, the contour offset had a significant effect on the support volume since as the contour offset increased, the support volume decreased significantly.

**Figure 21:**
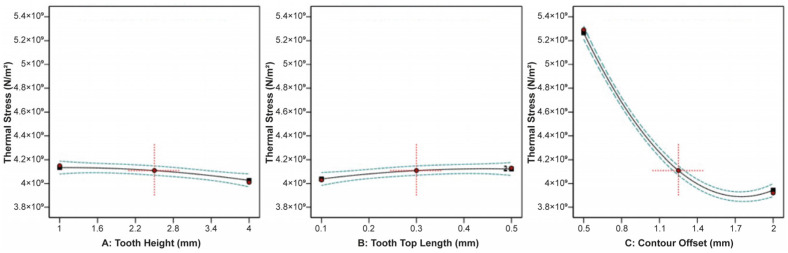
Main plots of thermal stress in Design-Expert 13 for contour-type supports.

**Figure 22:**
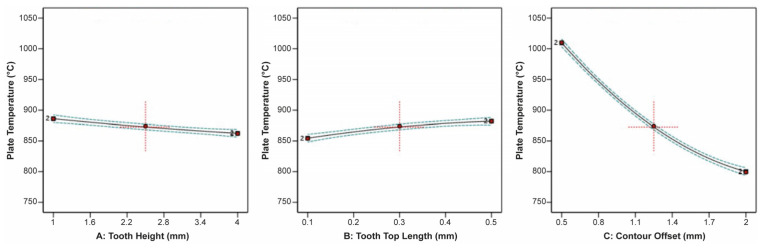
Main plots of plate temperature in Design-Expert 13 for contour-type supports.

The effect of the tooth height, tooth top length, and contour offset on the thermal stress applied on the build plate and the plate temperature are shown in Figure 21 and Figure 22, respectively. It was found that the tooth height and tooth top length did not significantly affect the thermal stress and plate temperature; however, it can be observed that as the tooth height increased, both the thermal stress and plate temperature slightly decreased, while as the tooth top length increased, the thermal stress and plate temperature slightly increased. Contrarily, the contour offset significantly affected the thermal stress and plate temperature since, as the offset increased, both the thermal stress and plate temperature greatly decreased.

**Figure 23:**
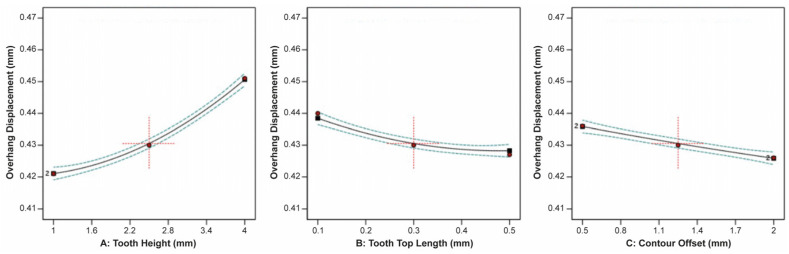
Main plots of overhang displacement in Design-Expert 13 for contour-type supports.

The effect of the tooth height, tooth top length, and contour offset on the overhang displacement is illustrated in Figure 23. From the plots, it is evident that all three input parameters had a substantial impact on the overhang displacement. When the tooth height increased, the overhang displacement increased significantly, but as both the tooth top length and contour offset increased, the overhang displacement decreased.

### 5.3. Cone Type

**Figure 24:**
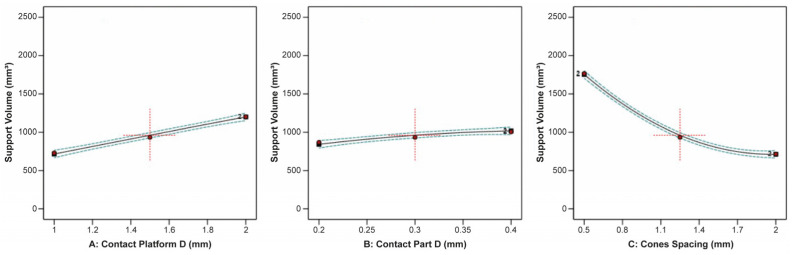
Main plots of support volume in Design-Expert 13 for cone-type supports.

Regarding the cone-type support structures, the findings displayed a degree of variation when compared to the block, line, and contour supports. This divergence arises from the distinct structure of cone-type supports, which consist of separate pillars with adjustable lower and upper diameters. Consequently, there was no tooth area defined by configurable tooth parameters in cone-type supports. In Figure 24, the effect of the contact platform diameter, contact part diameter, and cone spacing on the support volume is illustrated. It was found that an increase in the contact platform and contact part diameters led to a corresponding increase in the support volume. In contrast, an increase in the cone spacing resulted in a significant reduction in the support volume.

**Figure 25:**
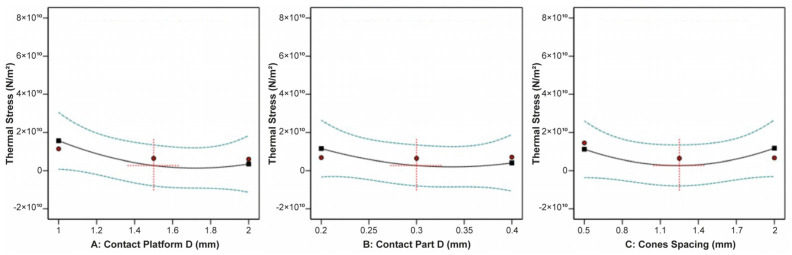
Main plots of thermal stress in Design-Expert 13 for cone-type supports.

The correlation between the contact platform diameter, contact part diameter, cone spacing, and the thermal stress applied on the build plate is shown in Figure 25. The plots indicated that none of the three input parameters had a significant impact on the thermal stress. However, the data revealed that the minimum thermal stress occurred at average values of the contact platform diameter, contact part diameter, and cone spacing.

**Figure 26:**
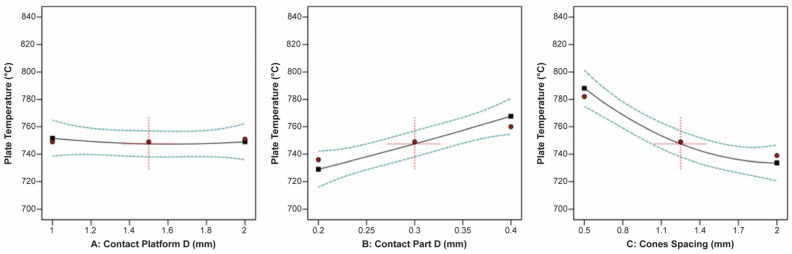
Main plots of plate temperature in Design-Expert 13 for cone-type supports.

On the other hand, the plate temperature was greatly affected by the contact part diameter and cone spacing, while the contact platform diameter barely affected the plate temperature. This is illustrated clearly in Figure 26. It was found that an increase in the contact part diameter led to a corresponding increase in the plate temperature, whereas an increase in the cone spacing resulted in a significant reduction in the plate temperature.

**Figure 27:**
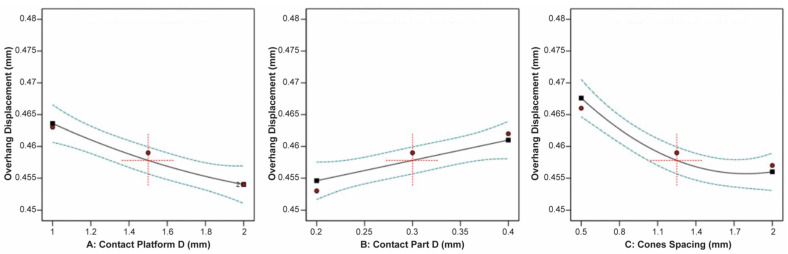
Main plots of overhang displacement in Design-Expert 13 for cone-type supports.

Regarding the specimen’s overhand displacement, it was greatly affected by the contact platform diameter, contact part diameter, and cone spacing. Based on the plots illustrated in Figure 27, it was noticed that as the contact platform diameter increased, the overhang displacement decreased, while as the contact part diameter increased, the displacement increased. Moreover, it was found that as the cone spacing increased, indicating a lower number of pillars, the overhang displacement decreased.

### 5.4. Numerical Optimisation for Block, Line, Contour, and Cone Supports

Following the analysis of the ANOVA results and the generation of correlation plots between the input variables and the output measures, numerical optimisation was performed in the Design Expert (v13) software for all four support types. The goal was to determine the optimal parameters for each support type, aiming to minimise the support volume, thermal stress, and overhang displacement, while maximising the plate temperature. The minimisation of the support volume was prioritised to reduce material consumption, resulting in decreased printing time and lower overall costs. Minimising the thermal stress on the build plate was crucial to prevent warping defects and ensure that the supports remained securely attached to the build plate. Similarly, minimising the overhang displacement was essential to maintain a high print quality and prevent defects like warping and curling. Conversely, the plate temperature was maximised since denser supports facilitate improved heat transfer and offer better control over residual stresses in the printed part [8,37].

To perform the numerical optimisation, a desirability approach was used. Desirability (D) evaluates how closely all the responses meet the assigned criteria and can range from 0 to 1. A “0” desirability score indicates that one or more responses fall outside the acceptable limits, while a “1” desirability score indicates that all the goals are perfectly satisfied [11]. The four support types: block, line, contour and cone, were studied separately using the same goals and criteria constraints as shown in Table 3.

**Figure 28:**
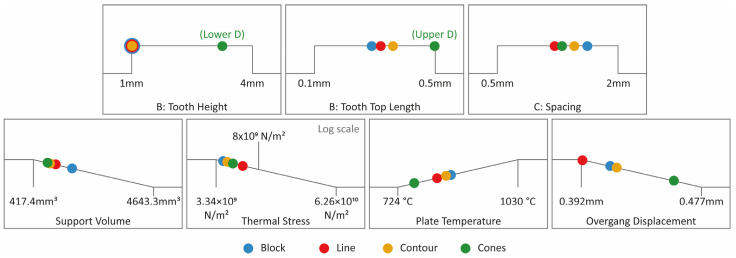
Graphical representation of optimal solutions for block, line, contour, and cone supports.

The optimal solutions, one for each support type according to the highest desirability, are shown in Table 4. Block-type supports are characterised by the lowest value of tooth height (1 mm), average values of tooth top length (0.295 mm), and average to high values of spacing (1.625 mm). Line-type supports are characterised by the lowest value of tooth height (1 mm), average values of tooth top length (0.347 mm), and average values of spacing (1.242 mm). Contour-type support structures are also characterised by the lowest value of tooth height (1 mm), average values of tooth top length (0.365 mm), and average values of spacing (1.402 mm). On the other hand, cone-type supports are characterised by average to high values of lower diameter (1.7 mm), the highest value of upper diameter, and average values of spacing (1.27 mm). The optimal solutions for block, line, contour and cone supports are illustrated clearly in Figure 28. The minimum support volume (1091 mm^2^) was found in cone supports, the minimum thermal stress (3.83 × 109 N/m^2^) and the maximum plate temperature (872.7 °C) in block supports, while the minimum overhang displacement (0.393 mm) was observed in line supports.

## 6. Discussion

Based on the numerical results, parameters related to the contact area between the part and supports, and those that define the geometry and density of the supports, play a major role in producing non-defective parts while simultaneously minimising both the printing time and overall costs. It was observed that block support structures effectively meet the optimisation criteria for reducing residual stresses and preventing part distortion, despite their relatively high volume. Subsequently, the findings were discussed and compared with previously published research. Further analyses were carried out on the four responses (support volume, thermal stress, plate temperature, and overhang displacement), and noteworthy observations made during the numerical simulations are presented.

### 6.1. Support Volume

**Figure 29:**
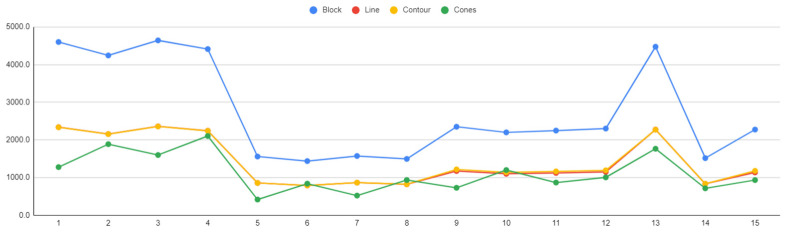
Support volume (mm^3^) vs. support alternatives.

Figure 29 illustrates the correlation between the support volume and the various alternatives of the four support types. As mentioned above in the Supplementary Materials files, based on the DOEs performed, alternatives 1–8 represent the factorial points, 9–14 are the axial points, and alternative 15 is the centre point. The distinction in the support density is evident, with block-type supports being significantly denser than line, contour, and cone supports, which exhibit similar density values. Additionally, it was noted that the plots for block, line, and contour supports demonstrate proportionality, given their closely aligned morphology. In contrast, the plot for cone-type supports displays a slight deviation. This variation is attributed to the unique structure of cone-type supports, which lack walls, specific grid patterns, or a tooth area. Instead, they consist of independent pillars with adjustable lengths and lower/upper diameters.

### 6.2. Thermal Stress

**Figure 30:**
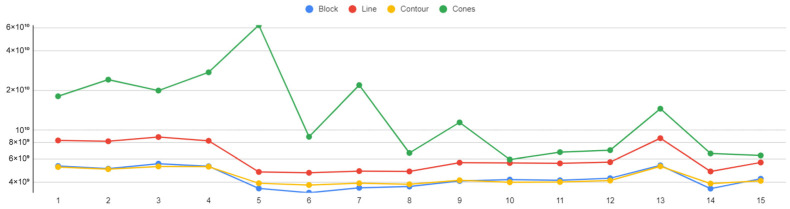
Thermal stress (N/mm²) vs. support alternatives (log scale).

The plots of the four support types that illustrate the correlation between the thermal stresses applied on the build plate and the support alternatives are shown in Figure 30. It was found that cone supports exhibited the greatest thermal stress on the build plate. Line-type supports also exhibited relatively high thermal stress values; whereas, block and contour support structures demonstrated the lowest levels of thermal stress.

**Figure 31:**
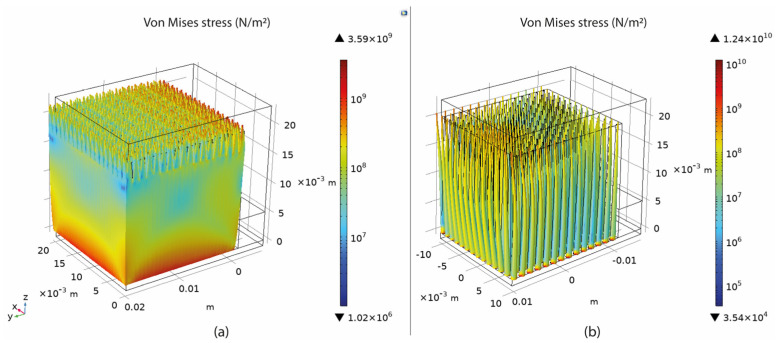
(**a**) Thermal stress on tooth area; (**b**) thermal stress on thin cone supports.

Based on the simulations, apart from the stresses that developed in the vicinity of the supports, a noteworthy amount of stress was observed on the top of the supports, particularly at the points where they contacted the printed part. The maximum stresses were observed on supports with high values of tooth height and low values of tooth top length (Figure 31a) and on cone-type supports with the lowest values of upper and lower diameters (Figure 31b). Such long and thin features are less able to deal with high laser temperatures and can collapse and affect the quality of the final part. This can be confirmed in the optimisation results shown in Figure 28, where such values were excluded.

### 6.3. Plate Temperature

**Figure 32:**
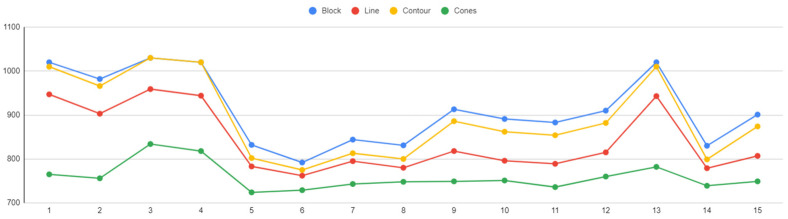
Plate temperature (°C) vs. support alternatives.

In Figure 32, the four plots for the measured temperature of the build plate for each support alternative are illustrated. The temperature span ranged from 724 °C to 1030 °C, with the highest values recorded in block-type supports and the lowest in cone-type supports.

**Figure 33:**
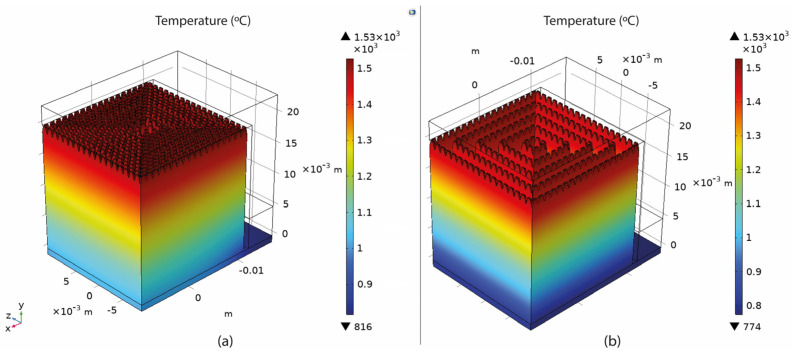
(**a**) Temperature distribution of high-density supports; (**b**) temperature distribution of low-density supports.

The connection between heat transfer and thermal stress is evident, as both factors influence the part’s performance during printing in LPBF at elevated temperatures. In this research, the temperature of the build plate was investigated to assess the effectiveness of supports in terms of optimal heat conduction. According to the simulations in COMSOL, it was found that higher build plate temperatures resulted in supports that better facilitated heat transmission. According to the optimisation findings, this is particularly achievable in high-density support structures. However, it is important to note that such high-density supports also experience higher levels of thermal stress. An example of the supports’ temperature distribution is shown clearly in Figure 33, where it can be observed that high-density supports (Figure 33a) resulted in a better temperature distribution than low-density supports (Figure 33b).

### 6.4. Overhang Displacement

**Figure 34:**
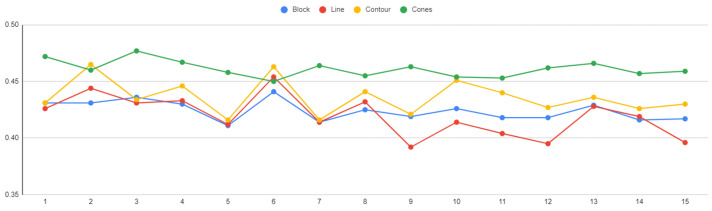
Overhang displacement (mm) vs. support alternatives.

The displacement plots of the part’s overhang surface for each alternative are shown in Figure 34. In this study, the tension of the part distorting under the high temperatures developed in LPBF was measured. The findings indicated that overhangs supported by cone-type support structures were more prone to warp, while block, bine, and contour support types had a similar impact on the overhang displacement of the part. As illustrated in the graph, the displacement ranged from 0.39 mm to 0.48 mm, with the lowest values observed in line-type support structures.

**Figure 35:**
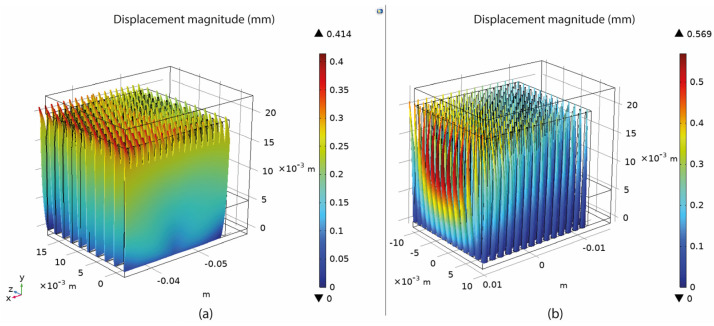
(**a**) Distortion of long teeth; (**b**) distortion of thin cone structures.

Another significant observation that occurred while conducting the thermo-mechanical simulations concerns the deformation of the supports under the high temperatures and thermal stresses developed around this area. As shown in Figure 35, it was found that elevated values of tooth height (e.g., 4 mm) and low values of lower/upper diameter (e.g., 1 mm/0.2 mm) led to supports that were more susceptible to distortion. It is worth noting that significant distortion in the main body of the supports and in the contact area between the overhang surface and the supports can potentially result in warping or, in more severe cases, print failure. The highest values of teeth deformation were found in line supports (Figure 35a), while very thin cones were those with the highest deformation (Figure 35b).

In addition to the geometric parameters of the supports examined in this study, there are other factors that significantly influence the distortion of an overhang surface during the printing process in laser-based technologies. These factors are associated with the heat source, such as laser speed and laser power [40]. These adjustable parameters not only impact part distortion but also affect the ease of support removal. For the purposes of this research, the heat source was kept constant to enhance the simulations. However, in future work, these parameters will be further investigated and evaluated through real-time experimentation.

### 6.5. Comparison of the Findings with Previously Published Work

Significant similarities were observed when comparing the findings of this research with relevant published work. The optimisation results showed that the optimum support density to maximise the thermo-mechanical performance and reduce the volume is characterised by average spacing values of 1.63 mm for the X, Y hatching for block supports, 1.24 mm for the cross line interval for line supports, 1.4 mm for the contour offset for contour supports, and 1.3 mm for the cone spacing for cone supports. Similar results were observed in previously published research (Dimopoulos et al.) [11] where, for 0° overhangs, block-type supports with a X, Y hatching equal to 2.5 mm were unable to be printed, while the optimum value was found to be 0.72 mm. In the same study, the optimal parameters for 0° overhangs regarding tooth height and tooth top length were found to be 2.74 mm and 0.23 mm, respectively. In this research, the optimal tooth height was found to be 1 mm and the optimal tooth top length to be 0.3 mm. Comparing the two studies, the tooth top length values are quite similar while tooth height values vary. This is because in the research of Dimopoulos et al., support removability was considered as a factor for the optimisation results. In this research, only the volume and the thermal behaviour of the supports were taken into consideration.

Similarities were also observed in Cheng et al. [8] where various support geometries, including block-type and lattice structures, were thermo-mechanically simulated and 3D printed. The study found that high-density block supports with an X, Y hatching of 0.45 mm resulted in a failed build due to high thermal stresses that caused significant warping on the ledge specimen. As a result, open-cell lattice structures with a higher density were proposed as an optimal solution, as they were better at relieving residual stresses and reducing part displacement. When comparing Cheng’s findings with the optimisation results of this study, it becomes evident that parameters leading to such high-density support structures (such as a spacing less than 1.24 mm) are excluded, as it was observed that the lowest values of support spacing were the ones that maximised thermal stresses and part displacement.

## 7. Conclusions

This study aimed to assess the performance of four common support structures (blocks, lines, contours, and cones) used in metal AM and LPBF. It involved 3D printing, the design of an experimental methodology, and thermo-mechanical simulations to examine the thermal behaviour of 60 distinct support configurations during laser-based 3D printing. The goal was to identify optimal solutions for each support type, ensuring cost-efficiency, minimal material usage, and the production of well-printed parts. The key objectives were to optimise the relief of residual stresses during printing, minimise part and support deformation, and reduce support volume. Based on the analysis and optimisation results, the most important conclusions are as follows:

- For block supports, the optimal solution was characterised by the lowest value of tooth height (1 mm), average values of tooth top length (0.295 mm), and average to high values of X, Y hatching (1.625 mm);
- For line supports, the optimal solution was characterised by the lowest value of tooth height (1 mm), average values of tooth top length (0.347 mm), and average values of cross line interval (1.242 mm);
- For contour support structures, the optimal solution was characterised by the lowest value of tooth height (1 mm), average values of tooth top length (0.365 mm), and average values of contour offset (1.402 mm);
- For cone supports, the optimal solution was characterised by average to high values of contact platform diameter (1.7 mm), the highest value of contact part diameter, and average values of cone spacing (1.27 mm);
- The average support volume of block-type supports was much higher (approx. 45–50% up) compared to line, contour, and cone support structures, which were based on the same input parameters;
- Higher thermal stresses were observed on high-density support structures. Also, supports with high values of tooth height and thin features such as thin cone supports were more exposed to high thermal stresses;
- High-density supports exhibited better temperature distribution compared to low-density support geometries;
- Overhangs supported by cone-type support structures were more prone to warp. Moreover, supports with high values of tooth height and low lower/upper diameter values were more prone to distort;
- The minimum support volume (1091 mm^3^) was found in cone supports, the minimum thermal stress (3.83 × 109 N/m^2^) and the maximum plate temperature (872.7 °C) in block supports, while the minimum overhang displacement (0.393 mm) in line support structures;
- In terms of the optimum thermal behaviour, block supports were those that better satisfied the optimisation criteria, despite their high volume.

Future work will include further research and experimentation to evaluate the findings regarding the proposed optimised support structures using 3D printing in LPBF. Similar test specimens and practical applications will be employed to assess the thermo-mechanical performance of the supports and the overall print quality. Furthermore, future work will emphasise an investigation into support removability. Developing supports that are easy to remove, minimise material consumption, and maintain high print quality is crucial in the context of metal additive manufacturing, contributing to the potential adoption of LPBF as a production method.

## Figures and Tables

**Table 1:** Block-type support parameters and levels.

Support Type	Parameter	Level 1	Level 2	Level 3
Block	Tooth Height (TH)	1 mm	2.5 mm	4 mm
	Tooth Top Length (TTL)	0.1 mm	0.3 mm	0.5 mm
	X, Y Hatching (XYH)	0.5 mm	1.25 mm	2 mm

**Table 2:** Line, contour, and cone support parameters and levels.

Support Type	Parameter	Level 1	Level 2	Level 3
Line	Tooth Height (TH)	1 mm	2.5 mm	4 mm
	Tooth Top Length (TTL)	0.1 mm	0.3 mm	0.5 mm
	Cross Line Interval (CLI)	0.5 mm	1.25 mm	2 mm
Contour	Tooth Height (TH)	1 mm	2.5 mm	4 mm
	Tooth Top Length (TTL)	0.1 mm	0.3 mm	0.5 mm
	Contour Offset (CO)	0.5 mm	1.25 mm	2 mm
Cones	Contact Platform Diameter			
	or Lower Diameter (LD)	1 mm	1.5 mm	2 mm
	Contact Part Diameter			
	or Upper Diameter (UD)	0.2 mm	0.3 mm	0.4 mm
	Cone Spacing (CS)	0.5 mm	1.25 mm	2 mm

**Table 3:** Goals and criteria constraints.

Type	Name	Goal	Lower Limit	Upper Limit
Block	Tooth Height	in range	1 mm	4 mm
	Tooth Top Length	in range	0.1 mm	0.5 mm
	X, Y Hatching	in range	0.5 mm	2 mm
Line	Tooth Height	in range	1 mm	4 mm
	Tooth Top Length	in range	0.1 mm	0.5 mm
	Cross Line Interval	in range	0.5 mm	2 mm
Contour	Tooth Height	in range	1 mm	4 mm
	Tooth Top Length	in range	0.1 mm	0.5 mm
	Contour Offset	in range	0.5 mm	2 mm
Cones	Contact Platform Diameter Contact Part Diameter Cone Spacing	in range	1 mm	2 mm
		in range	0.2 mm	0.4 mm
		in range	0.5 mm	2 mm
All	Support Volume	minimise	417.4 mm³	4643.3 mm³
All	Thermal Stress	minimise	3.34 × 10^9^ N/m²	6.26 × 10^10^ N/m²
All	Plate Temperature	maximise	724 °C	1030 °C
All	Overhang Displacement	minimise	0.392 mm	0.477 mm

**Table 4:** Optimum results of block, line, contour, and cone support structures.

Type	Tooth Height (mm)	Tooth Top Length (mm)	Spacing (mm)	Support Volume (mm^3^)	Thermal Stress (N/m²)	Plate Temperature (°C)	Overhang Displacement (mm)	Desirability
Block	1	0.295	1.625	1785	3.83 × 10^9^	872.7	0.414	0.679
Line	1	0.347	1.242	1196	5.75 × 10^9^	821.2	0.393	0.637
Contour	1	0.365	1.402	1094	4.02 × 10^9^	867.7	0.420	0.695
Cones	1.7 (LD)	0.4 (UD)	1.270	1091	4.42 × 10^9^	766.3	0.459	0.625

## Data Availability

Data are contained within the article and Supplementary Materials.

## Supplementary Materials

The following supporting information can be downloaded at: https://www.mdpi.com/article/10.3390/ma16227164/s1, The “DOE set-up” file.
