# Supplementary material for: Support Structures Optimisation for High-Quality Metal Additive Manufacturing with Laser Powder Bed Fusion: A Numerical Simulation Study
Source: Materials (Basel). 2023 Nov 14;16(22):7164. doi: 10.3390/ma16227164 (PMC10673092; doi:10.3390/ma16227164)
Supplement: Supplementary file 1 [file materials-16-07164-s001.zip › materials-2699048-supplementary.pdf]

1. Block Type - DOE

Std	Space Type	Factor 1<br>A: Tooth Height<br>(mm)	Factor 2<br>B: Tooth Top Length<br>(mm)	Factor 3<br>C: X, Y Hatching<br>(mm)	Response 1<br>Support Volume<br>(mm^3)	Response 2<br>Plate Temperature<br>(degC)	Response 3<br>Thermal Stress<br>($N/m^{2)}$	Response 4<br>Overhang Displacement<br>(mm)
1	Factorial	1	0,1	0,5	4600,8	1020	5,32E+09	0,431
2	Factorial	4	0,1	0,5	4243,3	982	5,08E+09	0,431
3	Factorial	1	0,5	0,5	4643,3	1030	5,54E+09	0,436
4	Factorial	4	0,5	0,5	4413,2	1020	5,3E+09	0,43
5	Factorial	1	0,1	2	1559,6	832	3,6E+09	0,411
6	Factorial	4	0,1	2	1438,4	792	3,34E+09	0,441
7	Factorial	1	0,5	2	1574	844	3,64E+09	0,414
8	Factorial	4	0,5	2	1496	831	3,72E+09	0,425
9	Axial	1	0,3	1,25	2350,2	913	4,1E+09	0,419
10	Axial	4	0,3	1,25	2200,8	891	4,2E+09	0,426
11	Axial	2,5	0,1	1,25	2248,5	883	4,15E+09	0,418
12	Axial	2,5	0,5	1,25	2302,5	910	4,3E+09	0,418
13	Axial	2,5	0,3	0,5	4475,2	1020	5,38E+09	0,429
14	Axial	2,5	0,3	2	1517	830	3,59E+09	0,416
15	Center	2,5	0,3	1,25	2275,5	901	4,26E+09	0,417

Support Volume – ANOVA for Quadratic model

Source	Sum of Squares	df	Mean Square	F-value	p-value	
**Model**	2,373E+07	9	2,637E+06	6946,07	< 0.0001	significant
A-Tooth Height	87647,04	1	87647,04	230,88	< 0.0001	
B-Tooth Top Length	11451,46	1	11451,46	30,17	0,0027	
C-X, Y Hatching	2,188E+07	1	2,188E+07	57628,51	< 0.0001	
AB	3638,05	1	3638,05	9,58	0,0270	
AC	18856,82	1	18856,82	49,67	0,0009	
BC	2464,02	1	2464,02	6,49	0,0514	
A^2	0,0003	1	0,0003	8,363E-07	0,9993	
B^2	0,0003	1	0,0003	8,363E-07	0,9993	
C^2	1,335E+06	1	1,335E+06	3517,25	< 0.0001	
**Residual**	1898,09	5	379,62			
**Cor Total**	2,373E+07	14				

Factor coding is **Coded**.
Sum of squares is **Type III – Partial**
The **Model F-value** of 6946,07 implies the model is significant. There is only a 0,01% chance that an F-value this large could occur due to noise.
P-values less than 0,0500 indicate model terms are significant. In this case A, B, C, AB, AC, C^2 are significant model terms. Values greater than 0.1000 indicate the model terms are not significant. If there are many insignificant model terms (not counting those required to support hierarchy), model reduction may improve your model.

__Plate Temperature – ANOVA for Quadratic model__

Source	Sum of Squares	df	Mean Square	F-value	p-value	
**Model**	93900,11	9	10433,35	5911,24	< 0.0001	significant
A-Tooth Height	1512,90	1	1512,90	857,17	< 0.0001	
B-Tooth Top Length	1587,60	1	1587,60	899,49	< 0.0001	
C-X, Y Hatching	88924,90	1	88924,90	50382,38	< 0.0001	
AB	378,13	1	378,13	214,24	< 0.0001	
AC	3,13	1	3,13	1,77	0,2408	
BC	1,13	1	1,13	0,6374	0,4609	
A^2	0,0714	1	0,0714	0,0405	0,8485	
B^2	82,57	1	82,57	46,78	0,0010	
C^2	1340,64	1	1340,64	759,57	< 0.0001	
**Residual**	8,82	5	1,76			
**Cor Total**	93908,93	14				

Factor coding is **Coded**.
Sum of squares is **Type III – Partial**
The **Model F-value** of 5911,24 implies the model is significant. There is only a 0,01% chance that an F-value this large could occur due to noise.
P-values less than 0,0500 indicate model terms are significant. In this case A, B, C, AB, B^2, C^2 are significant model terms. Values greater than 0.1000 indicate the model terms are not significant. If there are many insignificant model terms (not counting those required to support hierarchy), model reduction may improve your model.

__Thermal Stress – ANOVA for Quadratic model__

Source	Sum of Squares	df	Mean Square	F-value	p-value	
**Model**	7,998E+18	9	8,887E+17	93,92	< 0.0001	significant
A-Tooth Height	3,136E+16	1	3,136E+16	3,31	0,1283	
B-Tooth Top Length	1,020E+17	1	1,020E+17	10,78	0,0219	
C-X, Y Hatching	7,621E+18	1	7,621E+18	805,50	< 0.0001	
AB	1,445E+16	1	1,445E+16	1,53	0,2714	
AC	1,125E+16	1	1,125E+16	1,19	0,3253	
BC	5,000E+13	1	5,000E+13	0,0053	0,9449	
A^2	1,068E+16	1	1,068E+16	1,13	0,3366	
B^2	2,865E+14	1	2,865E+14	0,0303	0,8687	
C^2	1,882E+17	1	1,882E+17	19,89	0,0066	
**Residual**	4,731E+16	5	9,462E+15			
**Cor Total**	8,045E+18	14				

Factor coding is **Coded**.
Sum of squares is **Type III – Partial**
The **Model F-value** of 93,92 implies the model is significant. There is only a 0,01% chance that an F-value this large could occur due to noise.
P-values less than 0,0500 indicate model terms are significant. In this case B, C, C^2 are significant model terms. Values greater than 0.1000 indicate the model terms are not significant. If there are many insignificant model terms (not counting those required to support hierarchy), model reduction may improve your model.

Overhang Displacement – ANOVA for Quadratic model

Source	Sum of Squares	df	Mean Square	F-value	p-value	
**Model**	0,0010	9	0,0001	19,06	0,0023	significant
A-Tooth Height	0,0002	1	0,0002	28,95	0,0030	
B-Tooth Top Length	8,100E-06	1	8,100E-06	1,33	0,3011	
C-X, Y Hatching	0,0003	1	0,0003	41,02	0,0014	
AB	0,0001	1	0,0001	12,82	0,0159	
AC	0,0003	1	0,0003	45,31	0,0011	
BC	0,0000	1	0,0000	5,93	0,0590	
A^2	0,0001	1	0,0001	9,63	0,0267	
B^2	1,984E-07	1	1,984E-07	0,0326	0,8639	
C^2	0,0001	1	0,0001	9,63	0,0267	
**Residual**	0,0000	5	6,094E-06			
**Cor Total**	0,0011	14				

Factor coding is **Coded**.
Sum of squares is **Type III – Partial**
The **Model F-value** of 19,06 implies the model is significant. There is only a 0,23% chance that an F-value this large could occur due to noise.
P-values less than 0,0500 indicate model terms are significant. In this case A, C, AB, AC, A^2, C^2 are significant model terms. Values greater than 0.1000 indicate the model terms are not significant. If there are many insignificant model terms (not counting those required to support hierarchy), model reduction may improve your model.

2. Line Type - DOE

Std	Space Type	Factor 1<br>**A: Tooth Height**<br>(mm)	Factor 2<br>**B: Tooth Top Length**<br>(mm)	Factor 3<br>**C: Cross Line Interval**<br>(mm)	Response 1<br>**Support Volume**<br>(mm^3)	Response 2<br>**Plate Temperature**<br>(degC)	Response 3<br>**Thermal Stress**<br>(N/m^2)	Response 4<br>**Overhang Displacement**<br>(mm)
1	Factorial	1	0,1	0,5	2339,4	947	8,31E+09	0,426
2	Factorial	4	0,1	0,5	2157,6	903	8,19E+09	0,444
3	Factorial	1	0,5	0,5	2361	959	8,82E+09	0,431
4	Factorial	4	0,5	0,5	2244	944	8,26E+09	0,433
5	Factorial	1	0,1	2	857,8	783	4,8E+09	0,412
6	Factorial	4	0,1	2	791,1	762	4,73E+09	0,454
7	Factorial	1	0,5	2	865,7	795	4,87E+09	0,414
8	Factorial	4	0,5	2	822,8	780	4,84E+09	0,432
9	Axial	1	0,3	1,25	1175,1	818	5,64E+09	0,392
10	Axial	4	0,3	1,25	1100,4	796	5,61E+09	0,414
11	Axial	2,5	0,1	1,25	1124,3	789	5,57E+09	0,404
12	Axial	2,5	0,5	1,25	1151,3	815	5,69E+09	0,395
13	Axial	2,5	0,3	0,5	2275,5	943	8,64E+09	0,428
14	Axial	2,5	0,3	2	834,4	779	4,84E+09	0,419
15	Center	2,5	0,3	1,25	1137,8	807	5,66E+09	0,396

Support Volume – ANOVA for Quadratic model

Source	Sum of Squares	df	Mean Square	F-value	p-value	
**Model**	5,805E+06	9	6,450E+05	5867,22	< 0.0001	significant
A-Tooth Height	23338,56	1	23338,56	212,31	< 0.0001	
B-Tooth Top Length	3048,52	1	3048,52	27,73	0,0033	
C-Cross Line Interval	5,192E+06	1	5,192E+06	47233,34	< 0.0001	
AB	981,25	1	981,25	8,93	0,0305	
AC	4474,58	1	4474,58	40,71	0,0014	
BC	584,82	1	584,82	5,32	0,0692	
A^2	0,0039	1	0,0039	0,0000	0,9955	
B^2	0,0003	1	0,0003	2,888E-06	0,9987	
C^2	4,475E+05	1	4,475E+05	4070,79	< 0.0001	
**Residual**	549,63	5	109,93			
**Cor Total**	5,805E+06	14				

Factor coding is **Coded**.
Sum of squares is **Type III – Partial**
The **Model F-value** of 5867,22 implies the model is significant. There is only a 0,01% chance that an F-value this large could occur due to noise.
P-values less than 0,0500 indicate model terms are significant. In this case A, B, C, AB, AC, C^2 are significant model terms. Values greater than 0.1000 indicate the model terms are not significant. If there are many insignificant model terms (not counting those required to support hierarchy), model reduction may improve your model.

Plate Temperature – ANOVA for Quadratic model

Source	Sum of Squares	df	Mean Square	F-value	p-value	
**Model**	76298,00	9	8477,56	454,14	< 0.0001	significant
A-Tooth Height	1368,90	1	1368,90	73,33	0,0004	
B-Tooth Top Length	1188,10	1	1188,10	63,65	0,0005	
C-Cross Line Interval	63520,90	1	63520,90	3402,80	< 0.0001	
AB	153,13	1	153,13	8,20	0,0352	
AC	66,13	1	66,13	3,54	0,1186	
BC	66,13	1	66,13	3,54	0,1186	
A^2	4,96	1	4,96	0,2657	0,6282	
B^2	33,53	1	33,53	1,80	0,2378	
C^2	7888,96	1	7888,96	422,61	< 0.0001	
**Residual**	93,34	5	18,67			
**Cor Total**	76391,33	14				

Factor coding is **Coded**.
Sum of squares is **Type III – Partial**
The **Model F-value** of 454,14 implies the model is significant. There is only a 0,01% chance that an F-value this large could occur due to noise.
P-values less than 0,0500 indicate model terms are significant. In this case A, B, C, AB, C^2 are significant model terms. Values greater than 0.1000 indicate the model terms are not significant. If there are many insignificant model terms (not counting those required to support hierarchy), model reduction may improve your model.

Thermal Stress – ANOVA for Quadratic model

Source	Sum of Squares	df	Mean Square	F-value	p-value	
**Model**	3,647E+19	9	4,052E+18	331,73	< 0.0001	significant
A-Tooth Height	6,561E+16	1	6,561E+16	5,37	0,0683	
B-Tooth Top Length	7,744E+16	1	7,744E+16	6,34	0,0533	
C-Cross Line Interval	3,291E+19	1	3,291E+19	2693,92	< 0.0001	
AB	2,000E+16	1	2,000E+16	1,64	0,2568	
AC	4,205E+16	1	4,205E+16	3,44	0,1227	
BC	2,000E+16	1	2,000E+16	1,64	0,2568	
A^2	1,162E+16	1	1,162E+16	0,9513	0,3742	
B^2	9,956E+15	1	9,956E+15	0,8150	0,4080	
C^2	2,823E+18	1	2,823E+18	231,11	< 0.0001	
**Residual**	6,107E+16	5	1,221E+16			
**Cor Total**	3,653E+19	14				

Factor coding is **Coded**.
Sum of squares is **Type III – Partial**
The **Model F-value** of 331,73 implies the model is significant. There is only a 0,01% chance that an F-value this large could occur due to noise.
P-values less than 0,0500 indicate model terms are significant. In this case C, C^2 are significant model terms. Values greater than 0.1000 indicate the model terms are not significant. If there are many insignificant model terms (not counting those required to support hierarchy), model reduction may improve your model.

Overhang Displacement – ANOVA for Quadratic model

Source	Sum of Squares	df	Mean Square	F-value	p-value	
**Model**	0,0046	9	0,0005	136,04	< 0.0001	significant
A-Tooth Height	0,0010	1	0,0010	275,56	< 0.0001	
B-Tooth Top Length	0,0001	1	0,0001	32,45	0,0023	
C-Cross Line Interval	0,0001	1	0,0001	25,45	0,0039	
AB	0,0002	1	0,0002	52,97	0,0008	
AC	0,0002	1	0,0002	52,97	0,0008	
BC	0,0000	1	0,0000	6,49	0,0514	
A^2	0,0001	1	0,0001	21,02	0,0059	
B^2	0,0000	1	0,0000	2,88	0,1506	
C^2	0,0017	1	0,0017	462,37	< 0.0001	
**Residual**	0,0000	5	3,776E-06			
**Cor Total**	0,0046	14				

Factor coding is **Coded**.
Sum of squares is **Type III – Partial**
The **Model F-value** of 136,04 implies the model is significant. There is only a 0,01% chance that an F-value this large could occur due to noise.
P-values less than 0,0500 indicate model terms are significant. In this case A, B, C, AB, AC, A^2, C^2 are significant model terms. Values greater than 0.1000 indicate the model terms are not significant. If there are many insignificant model terms (not counting those required to support hierarchy), model reduction may improve your model.

3. Contour Type - DOE

		Factor 1	Factor 2	Factor 3	Response 1	Response 2	Response 3	Response 4
**Std**	**Space Type**	**A: Tooth Height**	**B: Tooth Top Length**	**C: Contour Offset**	**Support Volume**	**Plate Temperature**	**Thermal Stress**	**Overhang Displacement**
		(mm)	(mm)	(mm)	(mm^3)	(degC)	(N/mm^2)	(mm)
1	Factorial	1	0,1	0,5	2336,4	1010	5,23E+09	0,431
2	Factorial	4	0,1	0,5	2155	966	5,04E+09	0,465
3	Factorial	1	0,5	0,5	2358	1030	5,28E+09	0,434
4	Factorial	4	0,5	0,5	2241,4	1020	5,26E+09	0,446
5	Factorial	1	0,1	2	858	802	3,94E+09	0,416
6	Factorial	4	0,1	2	792	775	3,82E+09	0,463
7	Factorial	1	0,5	2	866	813	3,94E+09	0,416
8	Factorial	4	0,5	2	824	800	3,87E+09	0,441
9	Axial	1	0,3	1,25	1217,4	886	4,15E+09	0,421
10	Axial	4	0,3	1,25	1140,1	862	4,01E+09	0,451
11	Axial	2,5	0,1	1,25	1164,8	854	4,03E+09	0,44
12	Axial	2,5	0,5	1,25	1192,8	882	4,13E+09	0,427
13	Axial	2,5	0,3	0,5	2272,7	1010	5,29E+09	0,436
14	Axial	2,5	0,3	2	835	799	3,92E+09	0,426
15	Center	2,5	0,3	1,25	1178,8	874	4,11E+09	0,43

Support Volume – ANOVA for Quadratic model

Source	Sum of Squares	df	Mean Square	F-value	p-value	
**Model**	5,669E+06	9	6,299E+05	6633,98	< 0.0001	significant
A-Tooth Height	23357,89	1	23357,89	246,01	< 0.0001	
B-Tooth Top Length	3097,60	1	3097,60	32,62	0,0023	
C-Contour Offset	5,167E+06	1	5,167E+06	54424,44	< 0.0001	
AB	985,68	1	985,68	10,38	0,0234	
AC	4512,50	1	4512,50	47,53	0,0010	
BC	578,00	1	578,00	6,09	0,0567	
A^2	0,0020	1	0,0020	0,0000	0,9965	
B^2	0,0013	1	0,0013	0,0000	0,9972	
C^2	3,617E+05	1	3,617E+05	3809,97	< 0.0001	
**Residual**	474,74	5	94,95			
**Cor Total**	5,669E+06	14				

Factor coding is **Coded**.
Sum of squares is **Type III – Partial**
The **Model F-value** of 6633,98 implies the model is significant. There is only a 0,01% chance that an F-value this large could occur due to noise.
P-values less than 0,0500 indicate model terms are significant. In this case A, B, C, AB, AC, C^2 are significant model terms. Values greater than 0.1000 indicate the model terms are not significant. If there are many insignificant model terms (not counting those required to support hierarchy), model reduction may improve your model.

Plate Temperature – ANOVA for Quadratic model

Source	Sum of Squares	df	Mean Square	F-value	p-value	
**Model**	1,166E+05	9	12960,49	1200,54	< 0.0001	significant
A-Tooth Height	1392,40	1	1392,40	128,98	< 0.0001	
B-Tooth Top Length	1904,40	1	1904,40	176,41	< 0.0001	
C-Contour Offset	1,096E+05	1	1,096E+05	10154,26	< 0.0001	
AB	288,00	1	288,00	26,68	0,0036	
AC	24,50	1	24,50	2,27	0,1923	
BC	180,50	1	180,50	16,72	0,0095	
A^2	6,22	1	6,22	0,5764	0,4820	
B^2	50,79	1	50,79	4,71	0,0822	
C^2	2642,29	1	2642,29	244,76	< 0.0001	
**Residual**	53,98	5	10,80			
**Cor Total**	1,167E+05	14				

Factor coding is **Coded**.
Sum of squares is **Type III – Partial**
The **Model F-value** of 1200,54 implies the model is significant. There is only a 0,01% chance that an F-value this large could occur due to noise.
P-values less than 0,0500 indicate model terms are significant. In this case A, B, C, AB, BC, C^2 are significant model terms. Values greater than 0.1000 indicate the model terms are not significant. If there are many insignificant model terms (not counting those required to support hierarchy), model reduction may improve your model.

Thermal Stress – ANOVA for Quadratic model

Source	Sum of Squares	df	Mean Square	F-value	p-value	
**Model**	5,180E+18	9	5,755E+17	712,12	< 0.0001	significant
A-Tooth Height	2,916E+16	1	2,916E+16	36,08	0,0018	
B-Tooth Top Length	1,764E+16	1	1,764E+16	21,83	0,0055	
C-Contour Offset	4,369E+18	1	4,369E+18	5405,95	< 0.0001	
AB	6,050E+15	1	6,050E+15	7,49	0,0410	
AC	5,000E+13	1	5,000E+13	0,0619	0,8135	
BC	6,050E+15	1	6,050E+15	7,49	0,0410	
A^2	2,146E+15	1	2,146E+15	2,66	0,1641	
B^2	2,146E+15	1	2,146E+15	2,66	0,1641	
C^2	6,329E+17	1	6,329E+17	783,07	< 0.0001	
**Residual**	4,041E+15	5	8,082E+14			
**Cor Total**	5,184E+18	14				

Factor coding is **Coded**.
Sum of squares is **Type III – Partial**
The **Model F-value** of 712,12 implies the model is significant. There is only a 0,01% chance that an F-value this large could occur due to noise.
P-values less than 0,0500 indicate model terms are significant. In this case A, B, C, AB, BC, C^2 are significant model terms. Values greater than 0.1000 indicate the model terms are not significant. If there are many insignificant model terms (not counting those required to support hierarchy), model reduction may improve your model.

Overhang Displacement – ANOVA for Quadratic model

Source	Sum of Squares	df	Mean Square	F-value	p-value	
**Model**	0,0032	9	0,0004	329,32	< 0.0001	significant
A-Tooth Height	0,0022	1	0,0022	2028,15	< 0.0001	
B-Tooth Top Length	0,0003	1	0,0003	240,83	< 0.0001	
C-Contour Offset	0,0003	1	0,0003	231,48	< 0.0001	
AB	0,0002	1	0,0002	224,07	< 0.0001	
AC	0,0001	1	0,0001	78,24	0,0003	
BC	4,500E-06	1	4,500E-06	4,17	0,0967	
A^2	0,0001	1	0,0001	67,72	0,0004	
B^2	0,0000	1	0,0000	19,11	0,0072	
C^2	2,857E-07	1	2,857E-07	0,2646	0,6289	
**Residual**	5,400E-06	5	1,080E-06			
**Cor Total**	0,0032	14				

Factor coding is **Coded**.
Sum of squares is **Type III – Partial**
The **Model F-value** of 329,32 implies the model is significant. There is only a 0,01% chance that an F-value this large could occur due to noise.
P-values less than 0,0500 indicate model terms are significant. In this case A, B, C, AB, AC, A^2, B^2 are significant model terms. Values greater than 0.1000 indicate the model terms are not significant. If there are many insignificant model terms (not counting those required to support hierarchy), model reduction may improve your model.

4. Cones Type - DOE

		Factor 1	Factor 2	Factor 3	Response 1	Response 2	Response 3	Response 4
**Std**	**Space Type**	**A: Contact Platform**	**B: Contact Part**	**C: Spacing**	**Support Volume**	**Plate Temperature**	**Thermal Stress**	**Overhang Displacement**
		D mm	D mm	mm	mm^3	degC	N/mm^2	mm
1	Factorial	1	0,2	0,5	1278,4	765	1,8E+10	0,472
2	Factorial	2	0,2	0,5	1887,8	756	2,41E+10	0,46
3	Factorial	1	0,4	0,5	1600,2	834	1,99E+10	0,477
4	Factorial	2	0,4	0,5	2104,1	818	2,74E+10	0,467
5	Factorial	1	0,2	2	417,4	724	6,26E+10	0,458
6	Factorial	2	0,2	2	839	729	8,86E+09	0,45
7	Factorial	1	0,4	2	522,5	743	2,19E+10	0,464
8	Factorial	2	0,4	2	935,2	748	6,67E+09	0,455
9	Axial	1	0,3	1,25	729,3	749	1,14E+10	0,463
10	Axial	2	0,3	1,25	1200,3	751	5,95E+09	0,454
11	Axial	1,5	0,2	1,25	868,6	736	6,77E+09	0,453
12	Axial	1,5	0,4	1,25	1006,1	760	7,01E+09	0,462
13	Axial	1,5	0,3	0,5	1766,1	782	1,45E+10	0,466
14	Axial	1,5	0,3	2	715,2	739	6,61E+09	0,457
15	Center	1,5	0,3	1,25	934,1	749	6,39E+09	0,459

Support Volume – ANOVA for Quadratic model

Source	Sum of Squares	df	Mean Square	F-value	p-value	
**Model**	3,625E+06	9	4,028E+05	617,59	< 0.0001	significant
A-Contact Platform	5,850E+05	1	5,850E+05	896,86	< 0.0001	
B-Contact Part	76895,36	1	76895,36	117,90	0,0001	
C-Spacing	2,712E+06	1	2,712E+06	4157,42	< 0.0001	
AB	1635,92	1	1635,92	2,51	0,1741	
AC	9730,13	1	9730,13	14,92	0,0119	
BC	14179,28	1	14179,28	21,74	0,0055	
A^2	28,19	1	28,19	0,0432	0,8435	
B^2	2433,20	1	2433,20	3,73	0,1113	
C^2	1,910E+05	1	1,910E+05	292,84	< 0.0001	
**Residual**	3261,15	5	652,23			
**Cor Total**	3,629E+06	14				

Factor coding is **Coded**.
Sum of squares is **Type III – Partial**
The **Model F-value** of 617,59 implies the model is significant. There is only a 0,01% chance that an F-value this large could occur due to noise.
P-values less than 0,0500 indicate model terms are significant. In this case A, B, C, AC, BC, C^2 are significant model terms. Values greater than 0.1000 indicate the model terms are not significant. If there are many insignificant model terms (not counting those required to support hierarchy), model reduction may improve your model.

Plate Temperature – ANOVA for Quadratic model

Source	Sum of Squares	df	Mean Square	F-value	p-value	
**Model**	13138,91	9	1459,88	30,82	0,0007	significant
A-Contact Platform	16,90	1	16,90	0,3568	0,5763	
B-Contact Part	3724,90	1	3724,90	78,64	0,0003	
C-Spacing	7398,40	1	7398,40	156,20	< 0.0001	
AB	6,13	1	6,13	0,1293	0,7338	
AC	153,13	1	153,13	3,23	0,1321	
BC	1081,12	1	1081,12	22,83	0,0050	
A^2	20,64	1	20,64	0,4358	0,5383	
B^2	1,79	1	1,79	0,0377	0,8537	
C^2	457,14	1	457,14	9,65	0,0267	
**Residual**	236,82	5	47,36			
**Cor Total**	13375,73	14				

Factor coding is **Coded**.
Sum of squares is **Type III – Partial**
The **Model F-value** of 30,82 implies the model is significant. There is only a 0,07% chance that an F-value this large could occur due to noise.
P-values less than 0,0500 indicate model terms are significant. In this case B, C, BC, C^2 are significant model terms. Values greater than 0.1000 indicate the model terms are not significant. If there are many insignificant model terms (not counting those required to support hierarchy), model reduction may improve your model.

Thermal Stress – ANOVA for Quadratic model

Source	Sum of Squares	df	Mean Square	F-value	p-value	
**Model**	2,725E+21	9	3,027E+20	5,03	0,0451	significant
A-Contact Platform	3,699E+20	1	3,699E+20	6,14	0,0560	
B-Contact Part	1,403E+20	1	1,403E+20	2,33	0,1875	
C-Spacing	7,508E+17	1	7,508E+17	0,0125	0,9154	
AB	1,991E+20	1	1,991E+20	3,31	0,1287	
AC	8,522E+20	1	8,522E+20	14,15	0,0131	
BC	2,891E+20	1	2,891E+20	4,80	0,0800	
A^2	1,217E+20	1	1,217E+20	2,02	0,2144	
B^2	6,675E+19	1	6,675E+19	1,11	0,3407	
C^2	1,973E+20	1	1,973E+20	3,28	0,1301	
**Residual**	3,012E+20	5	6,023E+19			
**Cor Total**	3,026E+21	14				

Factor coding is **Coded**.
Sum of squares is **Type III – Partial**
The **Model F-value** of 5,03 implies the model is significant. There is only a 4,51% chance that an F-value this large could occur due to noise.
P-values less than 0,0500 indicate model terms are significant. In this case AC is a significant model term. Values greater than 0.1000 indicate the model terms are not significant. If there are many insignificant model terms (not counting those required to support hierarchy), model reduction may improve your model.

Overhang Displacement – ANOVA for Quadratic model

Source	Sum of Squares	df	Mean Square	F-value	p-value	
**Model**	0,0007	9	0,0001	34,76	0,0006	significant
A-Contact Platform	0,0002	1	0,0002	97,42	0,0002	
B-Contact Part	0,0001	1	0,0001	43,30	0,0012	
C-Spacing	0,0003	1	0,0003	142,24	< 0.0001	
AB	1,250E-07	1	1,250E-07	0,0529	0,8273	
AC	3,125E-06	1	3,125E-06	1,32	0,3023	
BC	1,250E-07	1	1,250E-07	0,0529	0,8273	
A^2	2,571E-06	1	2,571E-06	1,09	0,3448	
B^2	0,0000	1	0,0000	0,0000	1.0000	
C^2	0,0000	1	0,0000	17,40	0,0087	
**Residual**	0,0000	5	2,365E-06			
**Cor Total**	0,0008	14				

Factor coding is **Coded**.
Sum of squares is **Type III – Partial**
The **Model F-value** of 34,76 implies the model is significant. There is only a 0,06% chance that an F-value this large could occur due to noise.
P-values less than 0,0500 indicate model terms are significant. In this case A, B, C, C^2 are significant model terms. Values greater than 0.1000 indicate the model terms are not significant. If there are many insignificant model terms (not counting those required to support hierarchy), model reduction may improve your model.
